# Cereal grain 3D point cloud analysis method for shape extraction and filled/unfilled grain identification based on structured light imaging

**DOI:** 10.1038/s41598-022-07221-4

**Published:** 2022-02-24

**Authors:** Zhijie Qin, Zhongfu Zhang, Xiangdong Hua, Wanneng Yang, Xiuying Liang, Ruifang Zhai, Chenglong Huang

**Affiliations:** 1 College of Engineering, Huazhong Agricultural University, Wuhan, 430070 People’s Republic of China; 2 National Key Laboratory of Crop Genetic Improvement, National Center of Plant Gene Research (Wuhan), Huazhong Agricultural University, Wuhan, 430070 People’s Republic of China; 3 College of Informatics, Huazhong Agricultural University, Wuhan, 430070 People’s Republic of China

**Keywords:** Plant sciences, Plant breeding

## Abstract

Cereals are the main food for mankind. The grain shape extraction and filled/unfilled grain recognition are meaningful for crop breeding and genetic analysis. The conventional measuring method is mainly manual, which is inefficient, labor-intensive and subjective. Therefore, a novel method was proposed to extract the phenotypic traits of cereal grains based on point clouds. First, a structured light scanner was used to obtain the grains point cloud data. Then, the single grain segmentation was accomplished by image preprocessing, plane fitting, region growth clustering. The length, width, thickness, surface area and volume was calculated by the specified analysis algorithms for grain point cloud. To demonstrate this method, experimental materials included rice, wheat and corn were tested. Compared with manual measurement results, the average measurement error of grain length, width and thickness was 2.07%, 0.97%, 1.13%, and the average measurement efficiency was about 9.6 s per grain. In addition, the grain identification model was conducted with 25 grain phenotypic traits, using 6 machine learning methods. The results showed that the best accuracy for filled/unfilled grain classification was 90.184%.The best accuracy for indica and japonica identification was 99.950%, while for different varieties identification was only 47.252%. Therefore, this method was proved to be an efficient and effective way for crop research.

## Introduction

Because of population explosion, global warming, and water shortages, we are facing severe challenges in agricultural production^1–3^. Cereals mainly including rice, wheat, corn, and sorghum have occupied a dominant position in the human’s food^4^, and cereal production is of great importance to the food security^5,6^. Cereal grain traits including grain shape, grain plumpness have performed direct influence on the final yield, and grain traits measurement are necessary for yield-related research^7^. Grain shape is a very important basis of grain classification, and plumpness is the criterion for judging the quality of rice varieties. Therefore the grain trait extraction is essential for cereal research^8^. However, the conventional method mainly depends on manual measurement, which is inefficient, labor-intensive and subjective. Therefore, it is urgent to develop a novel method for grain trait extraction with high throughput and high accuracy.

The measurement of rice grain size is of great significance in rice breeding and genetic research. With the rapid development of computer technology, machine vision has been applied in grain size measurement^9,10^. Tanabata et al.^11^ developed Smart-Grain software for high-throughput measurement of seed shape based on digital images and the open computer vision library (OpenCV). Ma et al.^12^ extracted the length and width information of rice grains based on the images taken by smart phones. Le et al.^13^ proposed a method to study the morphology of developing wheat grains based on X-ray μCT imaging technique. However, most of the researches focus on the 2D traits^14^, and it is not easy to obtain the 3D grain traits such as volume, surface area and thickness. Since the grain size are small, high quality and complete point cloud of which is needed. Point clouds obtained by binocular stereo vision, structure from motion and space carving are relatively sparse^15–18^, on the contrary the structured light imaging, an active three-dimensional vision technology, can obtain high-precision point clouds, which is widely used in industrial detection, reverse engineering and cultural relic protection^19^, and it provides an effective method for high precision analysis of cereal grain 3D traits.

The rice grain plumpness is one of the determinants in yield, which is of great importance to rice breeding. The number of filled grains per panicle is directly related to the crop yield^20^. Therefore, counting of filled and unfilled grains of a panicle is critical to judge the rice quality. Traditionally, grain counting is performed manually, which is labor-intensive, time-consuming and subjective. Manually, filled grain is distinguished from unfilled grain by water-based or wind-based methods^21,22^. To improve it, some automated methods were developed for identifying and counting the filled grain. Duan et al.^23^ proposed a method based on visible light imaging and soft X-ray imaging, which was expensive, and of radiation risk. Kumar et al.^24^ built an automated system for discriminating and counting filled and unfilled grains of a rice panicle based on thermal images. Since the system required to monitor the temperature after heating the grains, it was complicated and difficult to achieve high-throughput measurement. Therefore, it is urgent to develop a new method for the recognition of filled/unfilled grains, with high efficiency and low radiation risk.

In this study, cereal grain traits analysis method based on point cloud was proposed. The high-precision point cloud of grains are obtained by structured light scanner, and the specified algorithms and integrated user software were designed for automatic segmentation of the grain point clouds and 3D grain trait extraction. Finally, 25 grain traits were computed, based on which, the model for filled/unfilled grain identification was set up. In conclusion, our research demonstrated a novel method for grain 3D and plumpness information extraction with high throughput and high accuracy, which was definitely helpful to the rice breeding and genetic research.

## Material and methods

### Material

**Figure 1:**
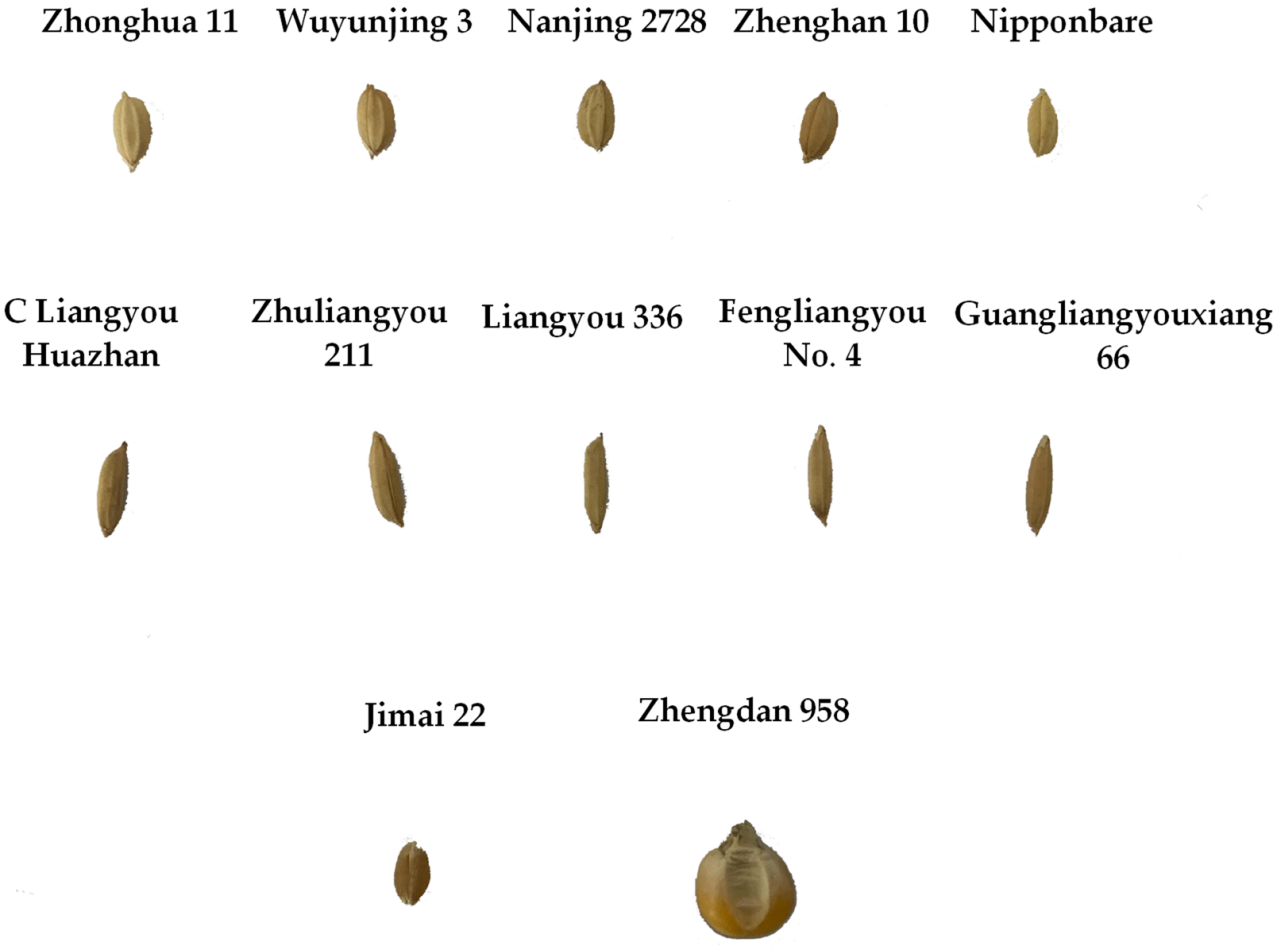
Display of experimental materials, including wheat grains, corn grains and 10 different varieties of rice grains.

In this study, the test materials included rice, wheat, and corn three types of cereals, which were purchased from the market and rice was the main part. 10 rice varieties including 5 indica and 5 japonica subspecies were selected. Each rice variety contained 100 filled grains and 100 unfilled grains, and a total of 2000 rice grains were used as experiment materials. In the filled and unfilled rice grains judgment, three experimenters would judge the same grain and the average judgment would be taken as ground truth. Moreover, 100 grains of wheat and corn were selected to validate the adaptability of this method. The experimental materials were shown as Fig. 1, and the rice experimental materials include Zhonghua 11, Wuyunjing 3, Nanjing 2728, Zhenghan 10, Nipponbare, C Liangyou Huazhan, Zhulaingyou 211, Liangyou 336, Fengliangyou No.4, Guangliangyouxiang 66. The first five varieties belonged to the rice subspecies of japonica, and the last five varieties belonged to the rice subspecies of indica.

### System design

#### 3D structured light scanner

**Figure 2:**
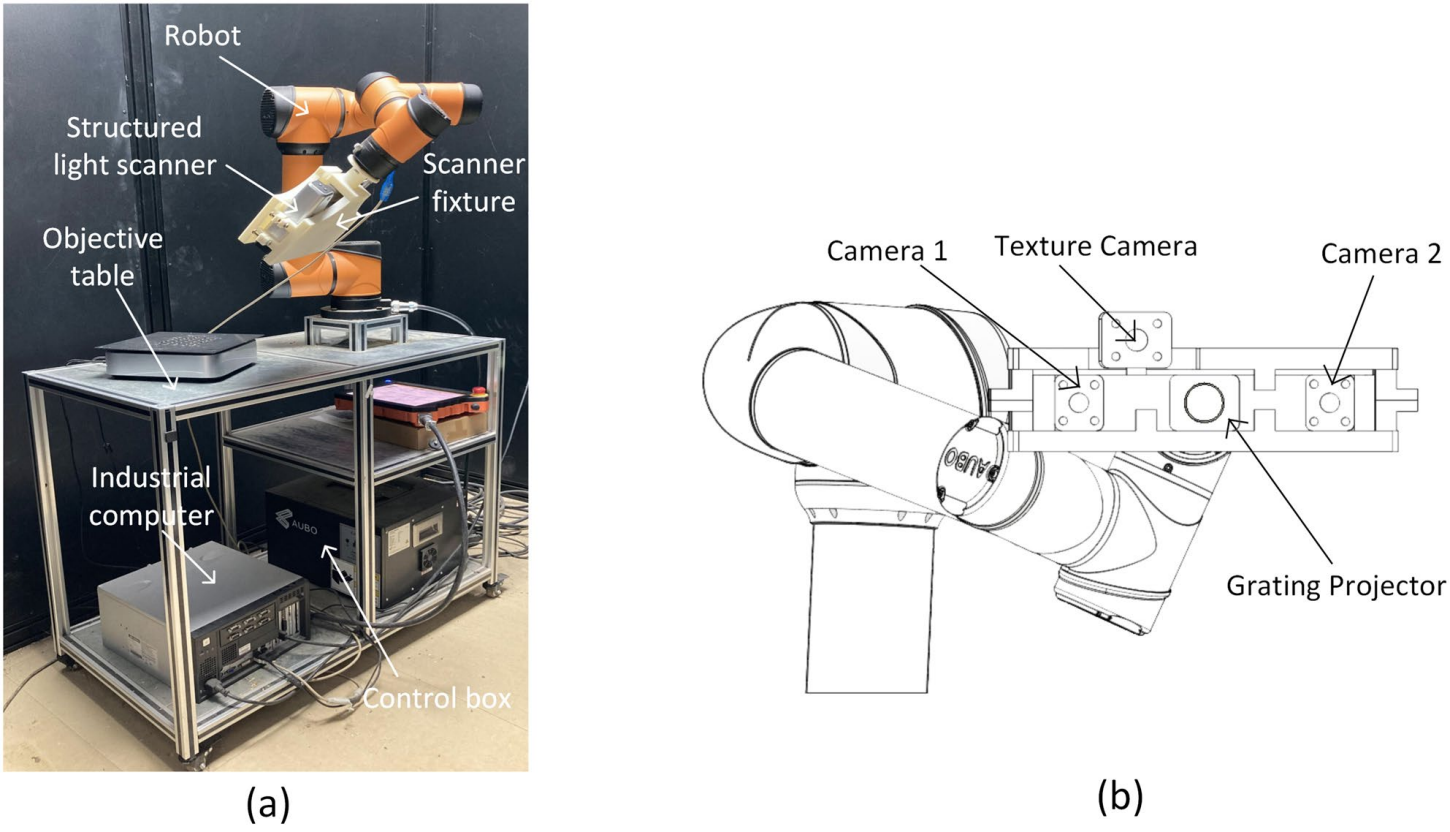
Schematic diagram of cereal grain scanning system. (**a**) The overall structure, (**b**) the structured light scanner.

The 3D structured light scanner (Reeyee Pro, China) was adopted in the study, which was based on white light LED raster scanning technology. Combing the advantages of structured light and binocular stereo vision, the scanner can achieve a single-sided accuracy of 0.05 mm within 2 s, which is suitable for high-precision scanning of small-sized work pieces, plastic products, and medical equipment. The main equipment is composed of a projector, two cameras and an internal modulated light source. Based on the principle of triangulation and sinusoidal grating image, it can obtain the dense point cloud data of objects. The detailed parameters of Reeyee Pro scanner are listed in Table 1. The structure of the scanner is shown in Fig. 2b.

**Table 1:** Reeyee Pro scanner detailed parameters.

Parameter	Value
Light source	White LED
Point distance	0.16 mm
Spatial resolution	0.05 mm
Scanning area	$210\times 150\mathrm{ mm}$
Working distance	290–480 mm
Maximum scan size	$200\times 200\times 200\mathrm{ mm}$

#### Cereal grain scanning system

As shown in Fig. 2a, the whole system consists of 6 parts: structured light scanner, robot, scanner fixture, object platform, industrial computer and control unit. AUBO i5 robot was adopted, which was a 6 degrees of freedom (DOF) collaborative robot with a positioning accuracy of ± 0.02 mm and a maximum load of 5 kg. The working range of the robotic arm was a sphere with a radius of 886.5 mm, which ensured sufficient scanning space. In order to fix the scanner on the robot, a fixture was designed and 3D printed with ABS material, and the entire weight of the scanner and the fixture was less than 2.5 kg. The object platform was designed to fix the robot and place samples. The industrial computer was connected with the control unit and the scanner, to achieve the cooperative operation of robot movement and the scanner imaging.

#### Cereal grain point cloud acquisition

**Figure 3:**
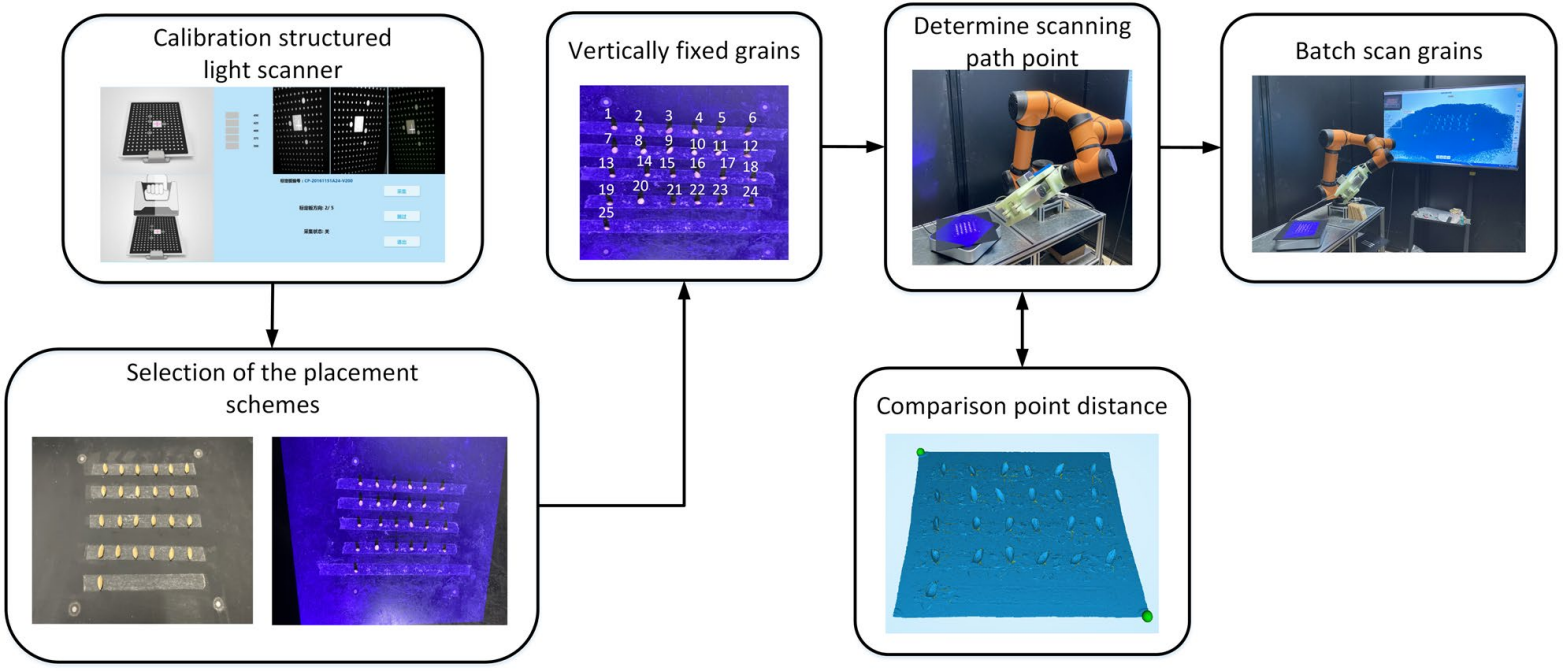
Flow chart of obtaining point cloud of cereal grains using structured light scanning system.

The cereal grain point cloud acquisition is shown as Fig. 3, which could be divided into 4 steps: the scanner calibration, the selection of the placement schemes, the scanning path determination, and the batch scanning.

1. Calibration of the scanner. The structured light scanner needed to be calibrated and corrected before working. When calibrating the camera, the calibration board need to be set in four positions including the directions of 0°, 90°, 180°, and 270°. Then the distance between the scanner and the calibration board should be adjusted from 350 to 450 mm, while collecting images.
2. Selection of the placement schemes. At present, there are mainly two kinds of three-dimensional scanning schemes for grains. One way is to spread the grains flatly on a platform, and another is to fix the grains through the seed holder^25^. The former has high efficiency, but the accuracy is low because the scanning grain is not complete. The latter obtains the complete point cloud of grain with high accuracy, but the disadvantage is that it can only scan a single grain, which is too time-consuming. To improve it, the grains were directly fixed vertically on the stage, and multiple grains could be scanned completely in the study.
3. Determination of the scanning path point. As shown in Table 1, the minimum space point distance of Reeyee Pro is 0.16 mm. To achieve as high spatial resolution as possible, the robot was studied to obtain proper scanning path point. In this study, the average minimum point distance of the grain point cloud was capable of reaching 0.1731 mm.
4. Batch scanning. Due to the limitation of scanning area and rotation effect, the grain placement range was set to $100\times 100\mathrm{ mm}$ in the center. In addition, the distance between adjacent grains was set as 20 mm to avoid grain shading. Meanwhile, the grain placement strategy was $6\times 4+1$ (4 rows for every 6 grains in a row, and the last one is placed separately), which is helpful for matching the manual and automatic values. What is more, the scanning strategy of rotating 8 times and scanning 45 degree a time was adopted.

#### System development environment

The configuration of the industrial computer is I5 3470 and GTX1050TI. The development environment is Windows 7 Pro, Visual Studio 2015, cross-platform open source Point Cloud library version 1.8.1(PCL) based on C++ (https://pointclouds.org), QT version 5.9.8 (https://www.qt.io), Python 3.7.6 and Visualization Toolkit version 8.0.0(VTK) (https://vtk.org). In addition, there is a software, Reeyee-Pro_V2.6.1.0 (https://www.wiiboox.net/support-software.php), which can display the 3D data collection of the point cloud in real time. And the robot is controlled by the Robot Operating System (ROS) system in the Ubuntu environment.

#### Cereal grain point cloud processing pipeline

**Figure 4:**
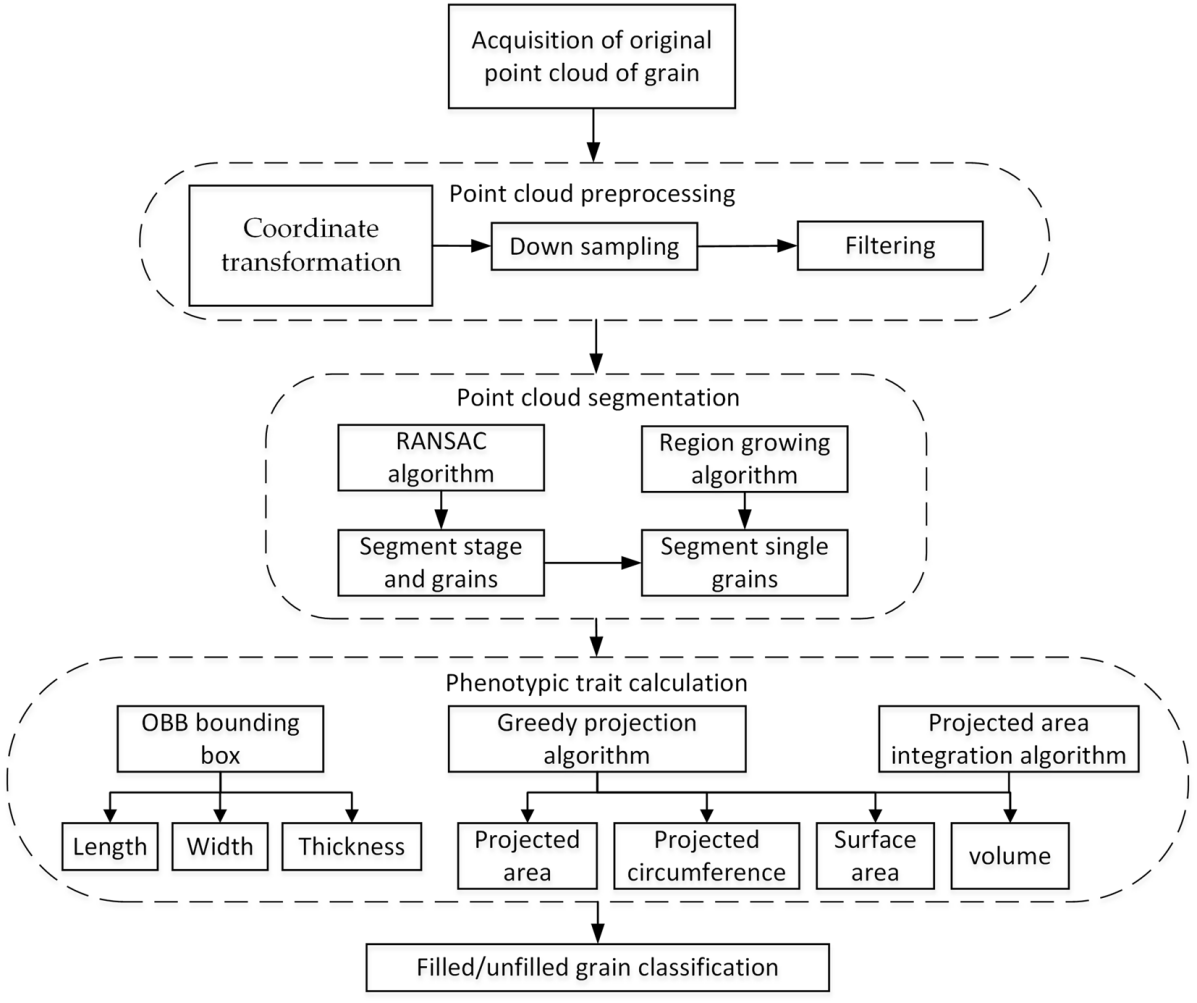
Cereal grain point cloud processing pipeline.

The overall processing pipeline of cereal grain point cloud is shown in Fig. 4. It mainly includes 4 steps: point cloud preprocessing, point cloud segmentation, phenotypic traits calculation, and filled/unfilled grain recognition.

### Preprocessing of point clouds

**Figure 5:**
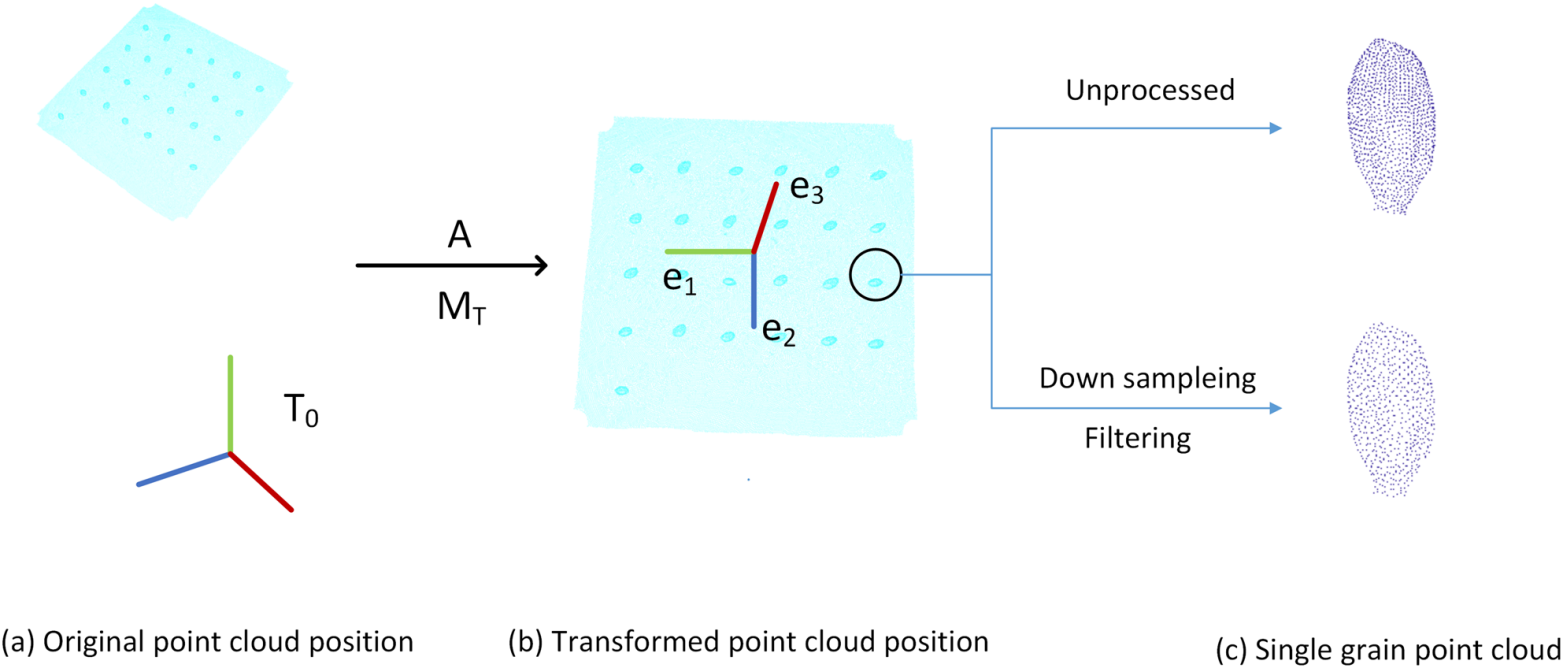
The process and result of preprocessing. (**a**) Original point cloud position, (**b**) transformed point cloud position, (**c**) single grain point cloud.

The preprocessing procedure of grain point cloud was shown in Fig. 5, mainly including 3 steps: coordinate transformation, down sampling, and filtering.

(1) The coordinate transformation were conducted as Eqs. (1, 2). Firstly, move the original coordinates (T0) to the centroid point of point cloud. Then, based on principal component analysis^26^ (PCA), the covariance matrix (MT) was computed to generate the new coordinate. The transformed result was shown as Fig. 5b.

1 $${T}_{A}={M}_{T}\times {(T}_{0}-A)$$

2 $${M}_{T}={\{{e}_{1},{e}_{2},{e}_{3}\}}^{T}$$

where ${e}_{1},{e}_{2},{e}_{3}$ are the three unit eigenvectors of the covariance matrix ${M}_{T}$; ${T}_{0}$ is the original point cloud coordinates; ${T}_{A}$ is the new coordinates after coordinate transformation; A is the translation matrix from the original coordinates (T0) to the centroid point of point cloud.

(2) Point cloud down sampling and filtering was shown as Fig. 5c. Based on voxel grids, all points in the voxel were replaced by the gravity center to reduce the point cloud, which can effectively improve the processing efficiency^27^. Then statistical filtering algorithm was applied to remove point data^28^, in which the point distance is abnormal.

#### Segmentation of point cloud

**Figure 6:**
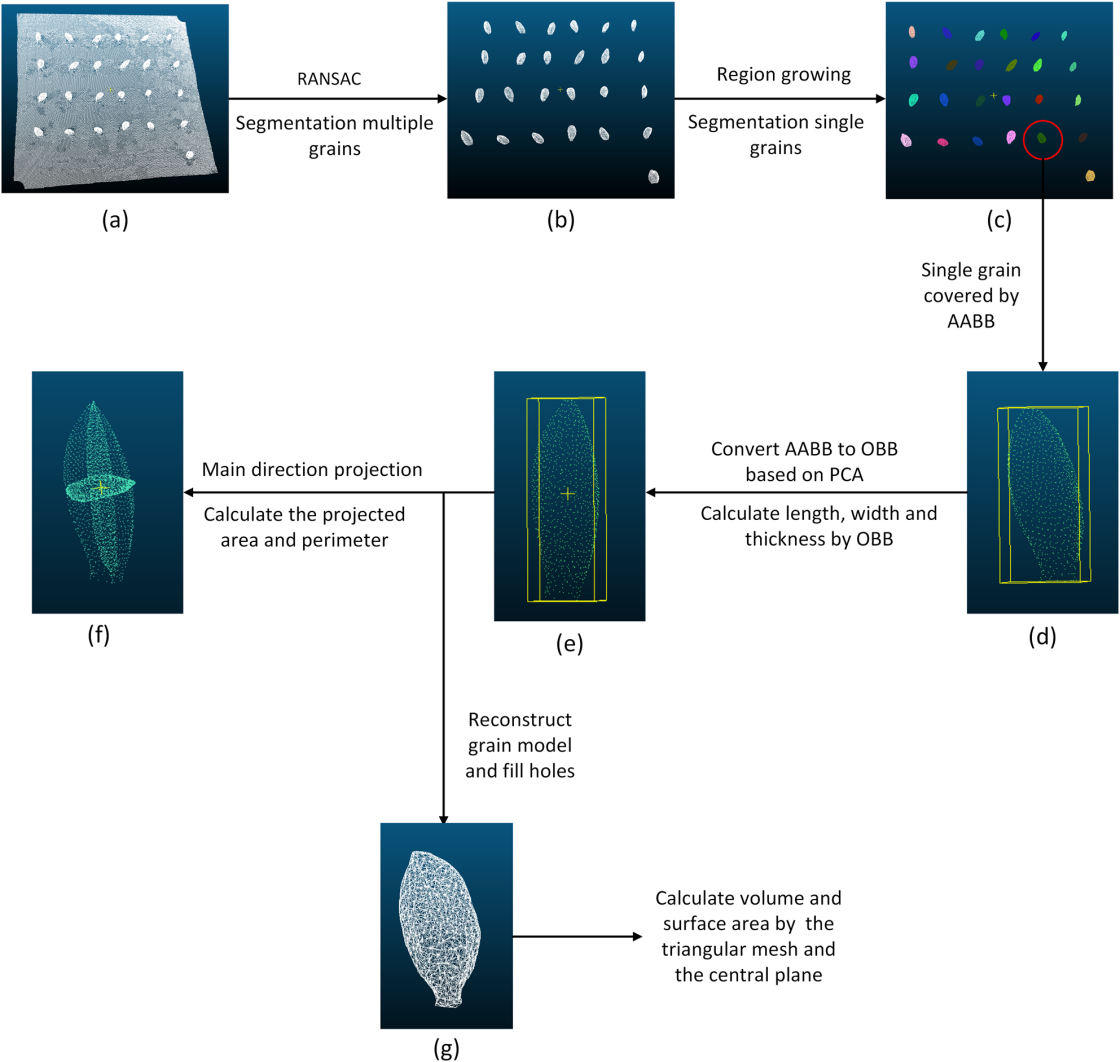
Cereal grain segmentation and traits extraction pipeline.

The segmentation of point cloud was conducted as Fig. 6. After the preprocessing, the random sample consensus algorithm (RANSAC) was adopted to fit the sample stage plane^29^ and separate the grain point clouds from the background. Then, based on curvature and normal angle, the single grain point cloud was identified by region growing algorithm^30^.

#### Phenotypic traits calculation

After single grain was obtained, phenotypic traits were extracted, including length, width, thickness, volume, surface area, projected area and perimeter in the main direction. Figure 6d–g shows the processing steps for grain trait extraction.

**Figure 7:**
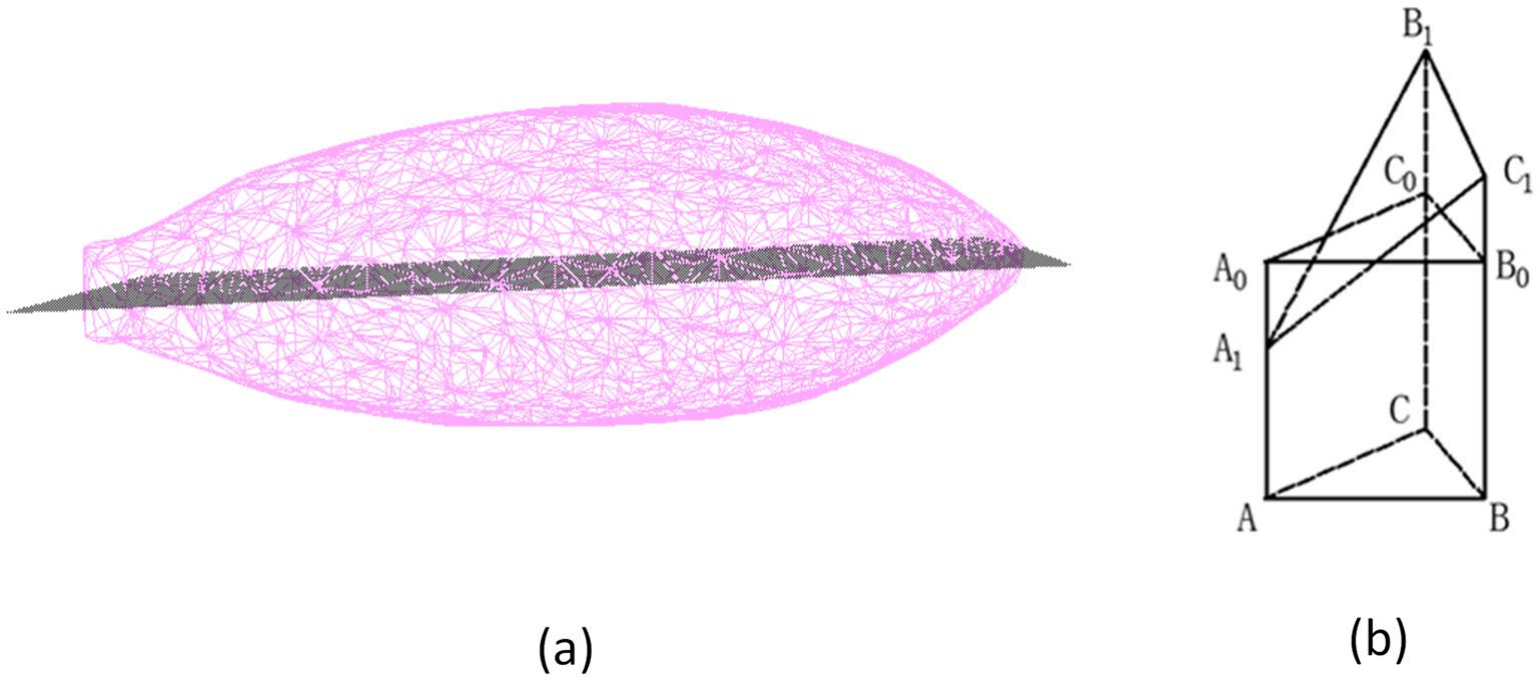
Grain volume calculation method in this study. (**a**) The central plane of triangular mesh projection, (**b**) the projected area integration method.

1. Grain length, width and thickness extraction
  As shown in Fig. 6d,e, the extraction of grain length, width and thickness was mainly achieved by constructing a bounding box. Firstly, the coordinate system of the segmented single grain point clouds were transformed to convert axis-aligned bounding box (AABB)^31^ into orientation bounding box (OBB)^32^. Secondly, the maximum and minimum values of the transformed single grain point cloud in the new coordinate system were calculated as ${x}_{max}, {x}_{min}, {y}_{max}, {y}_{min}, {z}_{max}, {z}_{min}$ respectively. Finally, the grain length, width and thickness were computed as following equations.
  3 $$l={x}_{max}-{x}_{min}$$
  4 $$w={y}_{max}-{y}_{min}$$
  5 $$h={z}_{max}-{z}_{min}$$
  where *l*, *w* and *h* are the length, width and thickness of a grain, respectively.
2. Grain surface area extraction
  Firstly, the triangular mesh model of the point clouds was established by greedy projection triangulation algorithm^33^.Secondly, the holes were filled by reconstructing the mesh boundary edges, which were generated by the grain segmentation. As shown in Fig. 6g, the length of the side of the triangle was calculated by the coordinates of the three vertices of the triangle. Then, based on Helen's formula^34^, the areas of all the triangular surfaces were calculated and the sum of them was used to approximate the surface area of the grain. The calculation formula is as Eqs. (6–7).
  6 $${S}_{0}=\sum_{i=1}^{k}{s}_{i}$$
  7 $${s}_{i}=\sqrt{{p}_{i}\left({p}_{i}-{a}_{i}\right)\left({p}_{i}-{b}_{i}\right)\left({p}_{i}-{c}_{i}\right)}$$
  where ${S}_{0}$ is surface area of a grain, *k* is total number of triangles, ${s}_{i}$ is area of the i-th triangle, ${p}_{i}$ is half the perimeter of the triangle, ${a}_{i}, {b}_{i} and {c}_{i}$ represent the length of each side of the triangle.
3. Grain volume extraction
  The grain volume was extracted as Fig. 7. Firstly, the convex pentahedrons were constructed by the triangular mesh and central plane projection, and then grain volume V was the sum of their volumes. Figure 7a is the central plane of the triangular mesh projection. And as shown in the Fig. 7b, ${A}_{1}, {B}_{1}\mathrm{ and }{C}_{1}$ are the three vertices of a triangular mesh. It is assumed that the volume of the straight triangular prism ${A}_{0}{B}_{0}{C}_{0}ABC$ is equal to the volume of this convex pentahedron, then the height of the straight triangular prism could be approximated as the height of the gravity center of ${\Delta A}_{1}{B}_{1}{C}_{1}$.
  8 $${V}_{{A}_{1}{B}_{1}{C}_{1}\mathrm{ABC}}={V}_{{A}_{0}{B}_{0}{C}_{0}\mathrm{ABC}}={S}_{\Delta ABC}\times \mathrm{h}\approx {S}_{\Delta ABC}\times {h}_{0}$$
  where $h$ is height of the straight prism, ${h}_{0}$ is height of the center of gravity of ${\Delta A}_{1}{B}_{1}{C}_{1}$
4. Projected area and perimeter of grain in the main direction extraction
  In this study, three main directions of grain point cloud were projected, and the projected area and perimeter of cross section, longitudinal section, and horizontal section were obtained as the shape description of grain (Fig. 6f). Firstly, the point cloud of a single grain after coordinate transformation was projected on the plane of x = 0, y = 0, z = 0 respectively. Then, based on the greedy projection triangulation algorithm^33^, the areas of the projected triangular mesh and the perimeter of the mesh edges were calculated.

#### Filled/unfilled grain analysis

A total of 25 phenotypic traits were extracted in the study, including 11 basic traits and 14 derived traits, as shown in Table 2. Compactness index, as a comprehensive grain shape description factor^35^, is calculated by the following formula:

9 $$c=\frac{{C}^{2}}{4\pi A}$$

where $\mathrm{c}$ is the compactness index, $C$ is perimeter of cross-section, $A$ is area of cross-section.

**Table 2:** 25 phenotypic traits.

No	Symbol	Trait	No	Symbol	Trait
1	$l$	Length	14	$w/h$	Width-thickness ratio
2	$w$	Width	15	${V}_{obb}$	Box volume
3	$h$	Thickness	16	$S/V$	Specific surface area
4	$V$	Volume	17	$S/l$	Surface area-length ratio
5	$S$	Surface area	18	$S/w$	Surface area-width ratio
6	${C}_{yz}$	Perimeter of cross section	19	$S/h$	Surface area-thickness ratio
7	${S}_{yz}$	Area of cross section	20	$V/l$	Volume-length ratio
8	${C}_{xz}$	Perimeter of longitudinal section	21	$V/w$	Volume-width ratio
9	${S}_{xz}$	Area of longitudinal section	22	$V/h$	Volume-thickness ratio
10	${C}_{xy}$	Perimeter of horizontal section	23	${c}_{yz}$	Compactness index of cross section
11	${S}_{xy}$	Area of horizontal section	24	${c}_{xz}$	Compactness index of longitudinal section
12	$l/w$	Length–width ratio	25	${c}_{xy}$	Compactness index of horizontal section
13	$l/h$	Length-thickness ratio			

With the rice grain phenotypic dataset, the models of recognition between filled and unfilled grains, distinction between indica and japonica subspecies, and classification of different rice varieties were established by six different machine learning algorithms including decision tree, random forest, support vector machine, Naive Bayes, XGBoost, and BP neural network^36–38^.

#### System software design

**Figure 8:**
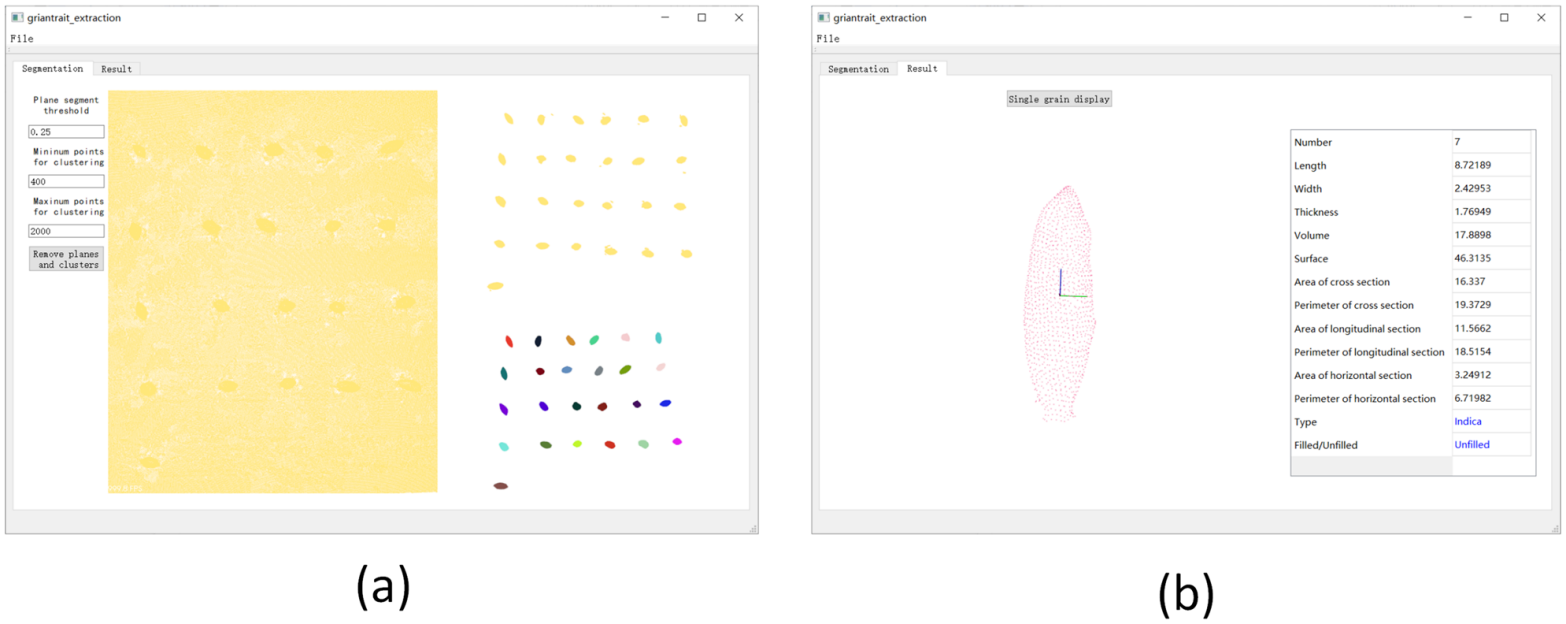
The user software for grain 3D point cloud analysis. (**a**) Grain 3D point cloud processing, (**b**) grain traits extraction.

In order to facilitate grain 3D point cloud analysis, A specific user software was designed based on QT Designer, PCL, QVTKWidget and XGBoost as shown in Fig. 8, in which the above algorithms including grain point cloud processing, grain traits calculation and analysis were integrated. The segmentation window displayed the original point cloud and the grain segmentation result as shown in Fig. 8a. Meanwhile, in order to predict the grain category and the plumpness, the python script was adopted to load the filled/unfilled grain classification model, and the result window displayed the single grain point cloud, 11 basic traits, categories and plumpness as shown in Fig. 8b. Moreover, the software parameters of plane segmentation threshold and cluster point cloud range were able to be easily modified by users to optimize the grain segmentation result. Finally the results including grain point cloud and traits would be saved, and the software operation was shown as Supplementary Video S1.

### Approval for plant experiments

We confirmed that all experiments were performed in accordance with relevant named guidelines and regulations.

## Results

To verify the accuracy of the algorithm, three experimenters used micrometers to measure the length, width and thickness of 2000 rice (including filled and unfilled grains), 100 wheat and 100 corn grains, and the mean value of the three measurements was taken as ground truth. The accuracy of the error analysis result is evaluated by mean absolute percentage error (MAPE), root mean square error (RMSE) and determination coefficient (${R}^{2}$). The relevant formula is as follows:

10 $$\mathrm{MAPE}=\frac{1}{n}\sum_{i}\frac{|{x}_{i}-{y}_{i}|}{{x}_{i}}\times 100\mathrm{\%}$$

11 $$\mathrm{RMSE}=\sqrt{\frac{{\sum }_{i}{({x}_{i}-{y}_{i})}^{2}}{n}}$$

12 $${R}^{2}=1-\frac{{\sum }_{i}{({x}_{i}-{y}_{i})}^{2}}{{\sum }_{i}{({x}_{i}-\overline{y })}^{2}}$$

where n is the total number of measurements; ${x}_{i}$ is the manual measurement results; ${y}_{i}$ is the system measurement results, and $\overline{y }$ is the mean of the system measurements.

### Comparison of placement scheme

**Figure 9:**
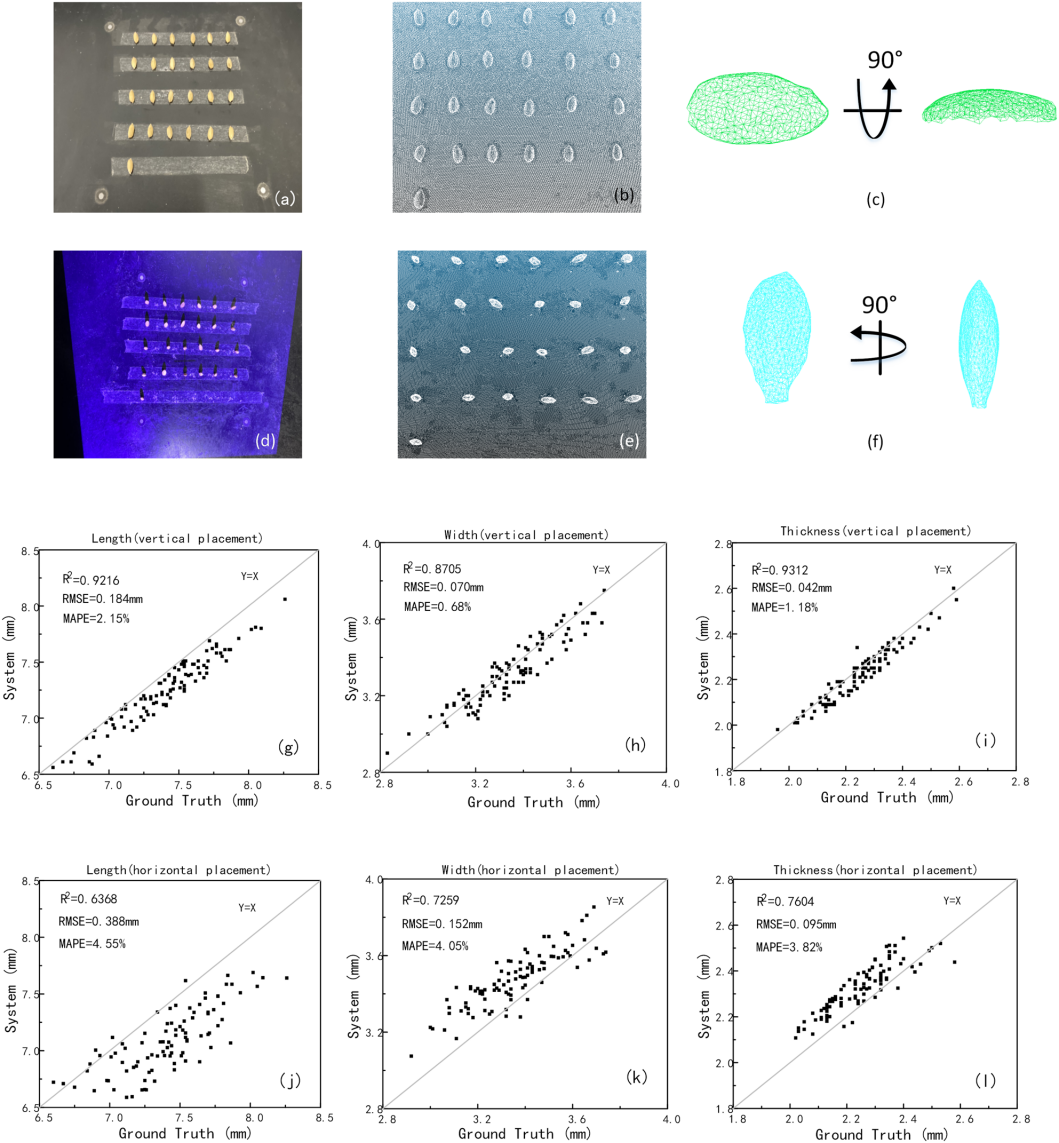
Comparison of two placement schemes, (**a**–**c**) the effect of horizontal placement scheme, (**d**–**f**) the effect of vertical placement scheme, (**g**–**i**) the measuring result in vertical placement, (**j**–**l**) the measuring result in horizontal placement.

To verify the measurement accuracy, 100 filled grains of Zhonghua 11 were taken as samples to compare the precision of the horizontal placement scheme with the vertical placement scheme. Figure 9a–f shows the point cloud comparison in the two schemes. As the results shown in Fig. 9g–l, the measurement errors of length, width and thickness of the horizontal placement scheme were 4.55%, 4.05% and 3.82%, while the measurement errors of the vertical placement scheme were 2.15%, 0.68% and 1.18%. As the Fig. 9c,f shown, the grain point clouds obtained by horizontal placement were incomplete due to the restriction of scanning angle, which obviously led to lower measurement accuracy, therefore the vertical placement scheme was proved to be preferable.

### Accuracy analysis for length, width, thickness, surface area and volume

**Figure 10:**
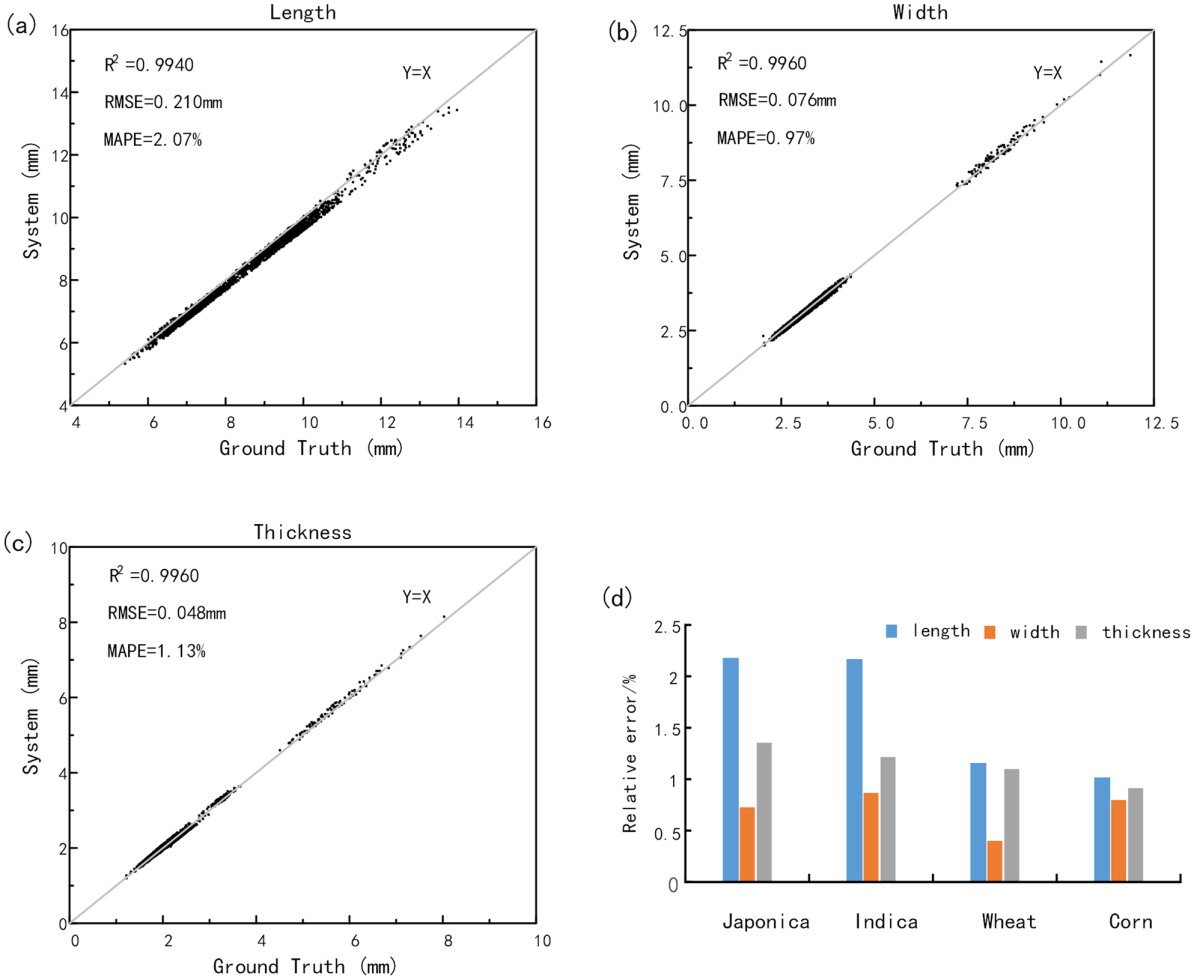
The sample accuracy analysis. (**a**) Length (**b**) Width (**c**) Thickness (**d**) japonica, indica, wheat and corn grains mean relative error.

Accuracy analysis was performed on all 2200 samples including rice, wheat and corn, and the measuring results were shown in Fig. 10. Figure 10a shows that the length measurement results of ${R}^{2},\mathrm{ RMSE},\mathrm{ MAPE}$ was 0.9940, 0.210 mm and 2.07% respectively. Figure 10b shows that the width measurement results of ${R}^{2},\mathrm{ RMSE},\mathrm{ MAPE}$ was 0.9960, 0.076 mm and 0.97% respectively. And Fig. 10c shows that the thickness measurement results of ${R}^{2},\mathrm{ RMSE},\mathrm{ MAPE}$ was 0.9960, 0.048 mm and 1.13% respectively. The results showed that the system value was in good consistency with the manual value and the system method was able to extract the grain length, width and thickness of grains with high precision. Meanwhile, as shown in Fig. 10d, the measurement errors of wheat and corn were generally smaller than rice, especially in the length, because the wheat and corn were more stable than rice when placed vertically, which led to higher scanning accuracy.

Due to the irregular surface morphology of the grains, the surface area and volume are difficult to measure in a non-destructive way. Therefore, a standard sphere with a radius of 10 mm was adopted to verify the system method validity. The results showed that the surface area and volume measuring error were 2.83% and 1.75% respectively.

### Statistical analysis of grain traits

The 25 grain traits extracted in this study could quantitatively describe the geometric shape of grain completely. In order to eliminate the influence of different dimensions of traits, the data was preprocessed based on the Z-score standardization method. The relevant formula is as follows:

13 $${X}^{*}=\frac{\mathrm{X}-\upmu }{\upsigma }$$

where X^*^ is the result of Z-score standardization, X is the sample data, $\upmu$ is the mean of sample data, $\upsigma$ is the standard deviation of sample data.

**Figure 11:**
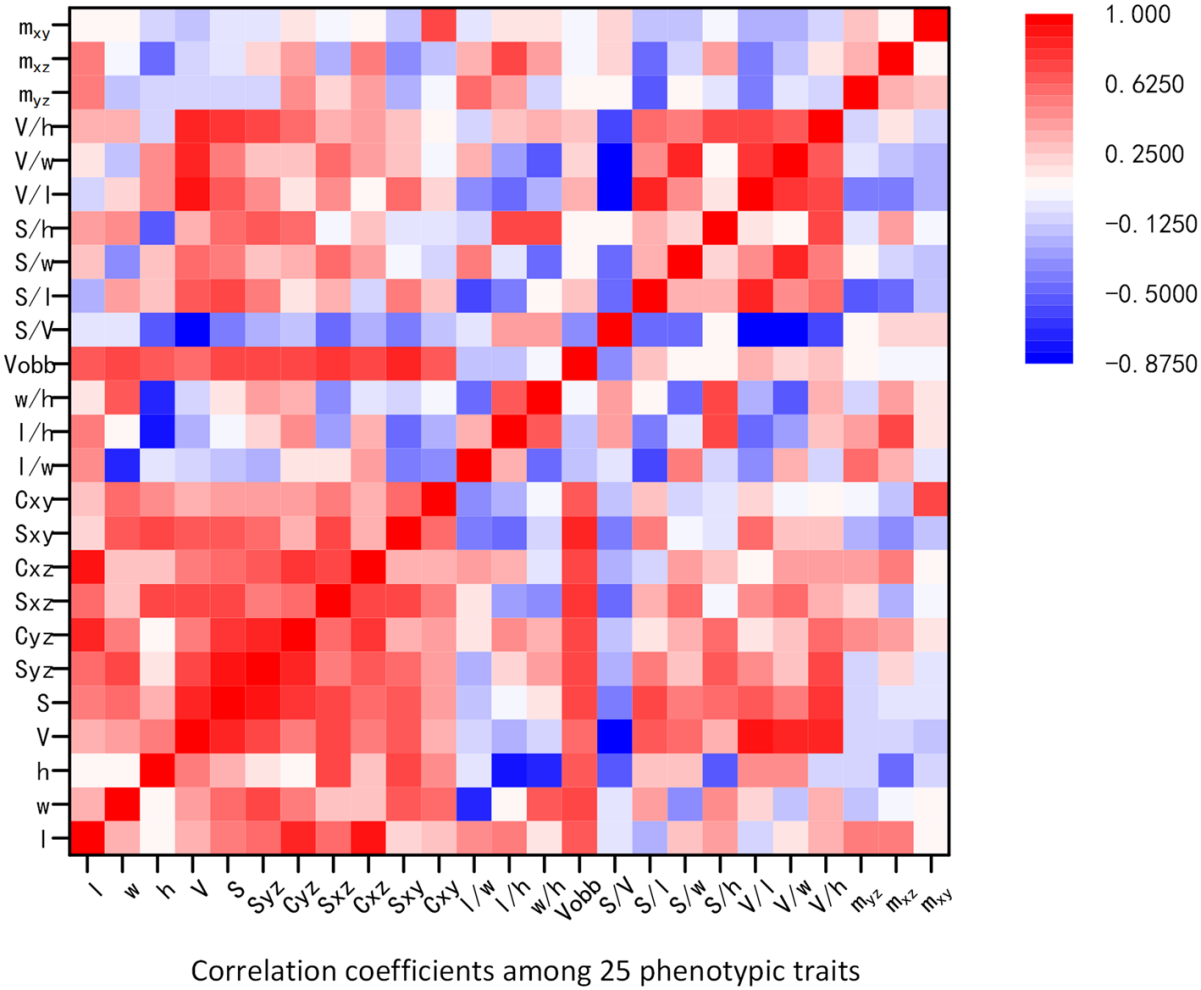
The result of grain traits correlation analysis.

Then the correlation analysis was carried out on the traits of grain varieties For example, with the extracted traits in Zhonghua 11, a correlation matrix of Pearson coefficients^38^ was calculated to identify inter-relationships. Intergroup correlation analysis was completed based on SPSS version 25.0 (https://www.ibm.com/products/spss-statistics), and the results were shown in Fig. 11. The results demonstrated that the correlation among the basic traits was strong and all of them were positive except thickness. Thickness as an important trait in grain shape had little correlation with length and width. In particular, the three compactness index were highly independent.

### Recognition model of filled and unfilled grains

Filled and unfilled grain identification has great importance to the finally yield evaluation. In this study, the classification models were studied by 6 different machine learning method with 25 phenotypic traits. All classification models were performed on the Sklearn Tool Kit version 0.24.2 (http://www.lfd.uci.edu/~gohlke/pythonlibs/#scikit-learn), and the main parameters were decided by learning curve and grid search method. Then tenfold cross-validation method was applied to validate each model. The data set was randomly divided into 10 parts, while 9 of them were taken as the training set in turn, and the rest as the test set. Then the average of the 10 results was used as the model's accuracy. The model results for filled and unfilled grains classification were shown in Table 3, the details of which was as follows:

1. Classification and regression trees (CART): The model was constructed as follows: the information entropy was set as impurity criterion. Meanwhile, the maximum tree depth was 4, and tree branch decision mode was random. The accuracy of model classification was 85.447%.
2. Random forest (RF): In this model test, the depth of the forest was set to 2, while the Gini coefficient was adopted, and the number of base evaluators was set to 24. According to the validation results, the model classification accuracy reached 88.605%. Compared with CART, the model accuracy was significantly improved.
3. Support vector machines (SVM): Since the distribution of original phenotypic traits is linearly inseparable, an optimal high-dimensional space was constructed by selecting the kernel function and the penalty factor. In this study, Gaussian kernel function was selected, and the penalty factor was set as 6. As a result, the accuracy of model classification was 89.684%.
4. Naive Bayes (NB): in this study, Gaussian Naive Bayes was selected and the classification accuracy rate was 88.079%.
5. Back propagation (BP) neural networks: The hidden layer was divided into two layers, in which the number of neurons in the first layer is 100 and the second layer is 50. The number of iterations was set to 2000, the initial learning rate was set to 0.0003237, and other parameters were the default values. Eventually, the classification accuracy of the model was 88.105%.
6. Extreme gradient boosting (XGBoost): The classifier was constructed based on tree model. After the logistic regression loss function was selected, the number of weak classifiers was set as 20, while the maximum tree depth was set as 5, and the learning rate was set as 0.3. As a result, the classification accuracy of the model was 90.184%, which was the best in all the models.

**Table 3:** The classification target results of each classification method based on 25 phenotypic traits.

Classification target	Method	Precision (%)	Recall score	F1 score
Filled and unfilled	CART	85.447	0.85333	0.85706
	RF	88.605	0.88722	0.89145
	SVM	89.684	0.89667	0.90371
	NB	88.079	0.88167	0.89363
	BP	88.105	0.88167	0.88811
	XGBoost	90.184	0.89333	0.90615
10 rice varieties	CART	37.027	0.36586	0.30779
	RF	40.363	0.39406	0.34210
	SVM	47.252	0.46856	0.44847
	NB	41.435	0.41175	0.39172
	BP	38.311	0.38047	0.36313
	XGBoost	45.960	0.45692	0.44967
Indica and japonica	CART	98.785	0.98750	0.98745
	RF	99.400	0.99400	0.99400
	SVM	99.950	0.99950	0.99950
	NB	99.450	0.99450	0.99450
	BP	99.250	0.99250	0.99245
	XGBoost	99.750	0.99950	0.99945

**Figure 12:**
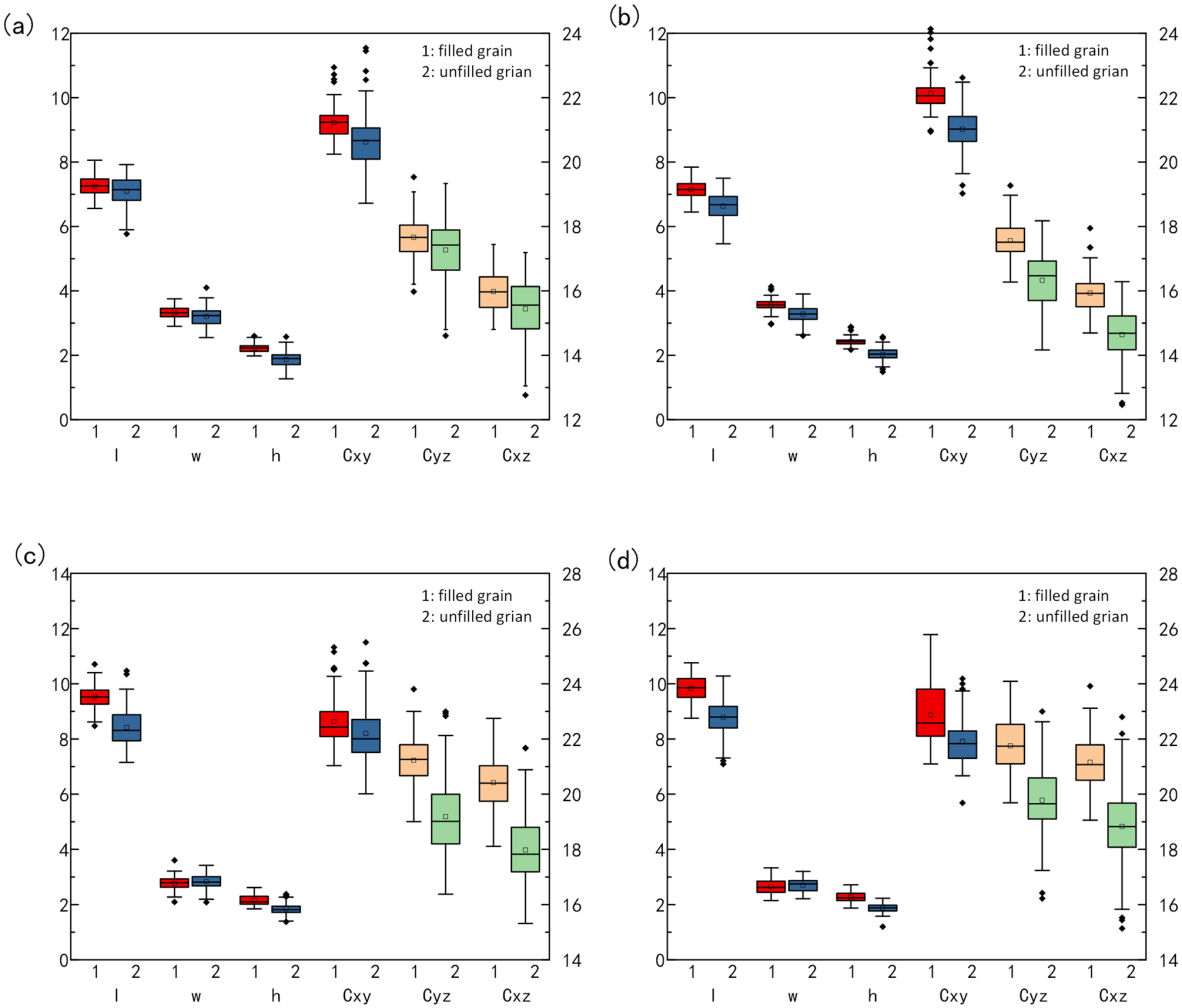
The Comparison of main traits between filled grain and unfilled grain. (**a**) Zhonghua 11 (**b**) Wuyunjing 3 (**c**) C Liangyou Huazhan (**d**) Zhuliangyou 211.

In order to explore the contribution of phenotypic traits, the XGBoost classifier was analyzed in detail and the results were shown in Table 4. From the results, the thickness weight had reached 0.34, which was proved to be dominant in filled and unfilled grain classification. Furthermore, the traits including volumetric-width ratio, volume, length-thickness ratio and surface area-length radio were all related to length, the weight of which were greater than 4%. Moreover, 4 varieties of rice grains were selected to verify the traits significance in the filled and unfilled grain classification. As shown in Fig. 12, the results indicated that the thickness had higher difference than width and length. The result also proved that the length had higher difference than width, especially in indica.

**Table 4:** Weight rank of characteristic traits (> 4%).

Rank	Trait	Importance weight
1	Thickness	0.342219
2	Length	0.067255
3	Perimeter of horizontal section	0.062472
4	Volume-width ratio	0.056376
5	Compactness index of horizontal section	0.053502
6	Volume	0.049749
7	Length-thickness ratio	0.042486
8	Surface area-length ratio	0.042199

### Classification of different rice varieties and classification of indica and japonica

Based on the same 6 machine learning methods, the grain phenotypic traits of 10 different varieties which belonged to the two subspecies of indica and japonica, were used to build classification model according to the tenfold cross-validation method. The results showed that the best performance for the different varieties classification was 47.252% by the SVM model, however the best performance for different subspecies classification was 99.950% by the SVM model. This is because the grain phenotypic traits in the same subspecies had much less difference than in different subspecies. The detailed classification results of different rice varieties and subspecies were shown in Table 3.

### Efficiency evaluation

To obtain the complete point clouds, 25 cereal grains would be scanned 8 times, and it took about 14 s for each time, while the sample turntable rotated 45 degree. Therefore, it took about 2 min for the point clouds acquisition. Meanwhile it took about 2 min for point clouds segmentation and phenotypic traits computation. Thus 25 grains measurement totally cost about 4 min, and the average efficiency was 9.6 s per grain. However the manual measurement efficiency was about 120 s per grain, which was one-twelfth of the system efficiency.

## Discussion

Cereal grain traits have important impact on the final yield, which are also necessary for crop breeding and genetic analysis. Phenotypic traits such as length, width, thickness, volume and surface area are of great significance. In this study, a novel method for grain trait extraction by 3D structured light imaging was invented with high-throughput and high-accuracy. In addition, the grain identification model was conducted with 25 grain phenotypic traits, using 6 machine learning methods. The results indicated that the thickness was dominant in filled and unfilled grain classification. The result also proved that the length had higher difference than width, especially in indica.

At present, distinguishing filled grain from unfilled grain mainly relies on water-based or wind-based methods which are inaccurate and destructive. There are few researches on the filled/unfilled grain distinction. Therefore, there is an urgent need for a method that can accurately identify filled and unfilled grains. Liu et al.^20^ designed a method based on image analysis to measure grain plumpness by the grain shadow in four directions. In addition, some methods were proposed based on X-ray and thermal imaging^23,24^, but all these methods were identified in 2D imaging and could not provide more phenotypic information. Hua et al.^25^ extracted the point cloud of rice grains based on a laser scanner to calculate phenotypic information. However, it was not suitable for requirements of high throughput. The method of this study can obtain the phenotypic information of grains with high precision and high efficiency, which provides a method for crop breeding research.

In the research of the placement method, it was confirmed that the vertical placement was more accurate than the horizontal placement. Also, it is worth noting that during the scanning process, the stability of the vertical placement played great effects on the measuring result. From the results, the measurement errors of wheat and corn were generally smaller than rice, especially in the length, because the wheat and corn were more stable than rice when placed vertically.

With the rice varieties and subspecies classification results, it is demonstrated that the performance for rice subspecies classification were much better than different rice varieties classification. In the parental research of rice material, it was found that the same rice subspecies had the same intersecting pedigrees. For example, the rice varies of Zhonghua 11 and Nipponbare, which both belong to the subspecies of japonica, had the same parant of Nonglin 22, and it would definitely lead to the relatively consistent phenotypic traits^40,41^. However the different subspecies would had few intersecting pedigrees, which would result in significant phenotypic traits difference.

## Conclusion

Based on the 3D structured light imaging, a novel method for cereal grain shape extraction and filled/unfilled grain identification was proposed. The results showed that the system measurement had high consistency with the manual measurement and the system method was able to extract the grain length, width and thickness of grains with high precision. Filled/unfilled grain identification, and grain subspecies classification were achieved by XGBoost and SVM Model, while a specific user software was developed to facilitate grain 3D point cloud analysis. In conclusion, our research demonstrated a novel method for grain 3D and plumpness information extraction with high throughput and high accuracy, which was definitely helpful to the rice breeding and genetic research. Based on the experiment results, the following conclusions are drawn.

1. Considering grain placement methods, the vertical placement scheme performed better results than the horizontal placement scheme. The measurement errors of length, width and thickness in the horizontal placement were 4.55%, 4.05% and 3.82%, while the measurement errors in the vertical placement were only 2.15%, 0.68% and 1.18%.
2. 25 phenotypic traits of cereal grains could obtained automatically in batch, including 11 basic traits, 14 derived traits. And the average efficiency for single grain measurement was about 9.6 s, including 3D structure light imaging and point clouds analysis.
3. 2200 samples including rice, corn and wheat were tested to evaluate this method, and the results showed that the average relative errors of length, width and thickness were 2.07%, 0.97% and 1.13%.
4. With the extracted traits, a correlation matrix of Pearson coefficients was calculated to identify inter-relationships. The results demonstrated that thickness as an important trait in grain shape had little correlation with length and width. In particular, the three compactness index were highly independent.
5. 6 machine learning methods were used to classify the phenotypic traits of the filled/unfilled grains of 10 kinds of grains. The results showed that XGBoost was the best in all the models, with the classification accuracy of the model was 90.184%, while the thickness was proved to be dominant in filled and unfilled grain classification. And for the classification among 10 different varieties of rice grains, the best performance was 47.252% by the SVM model. What’s more, all the models performed great to classify indica and japonica, and the best performance was 99.950 by the SVM model.

## Supplementary Information

Supplementary Information 1.

Supplementary Information 2.

Supplementary Information 3.

Supplementary Information 4.

Supplementary Information 5.

Supplementary Video 1.

## Data Availability

All data generated or analyzed during this study are included in this published article and its supplementary information files.
