# Supplementary material for: Cereal grain 3D point cloud analysis method for shape extraction and filled/unfilled grain identification based on structured light imaging
Source: Sci Rep. 2022 Feb 24;12:3145. doi: 10.1038/s41598-022-07221-4 (PMC8873360; doi:10.1038/s41598-022-07221-4)
Supplement: Supplementary file 1 — Supplementary Information 1. [file 41598_2022_7221_MOESM1_ESM.docx]

**Cereal grain 3D point cloud analysis method technical documentation**

**Detailed methodological information**

1. **Coordinate transformation**

By using the rotation and translation matrix to correct the coordinate system, the centroid of the original point cloud was converted to the origin of the coordinate system. The main steps of the conversion were as follows: First calculate the centroid coordinates of the point cloud model, and move the origin of the initial coordinate system to the point to form a new point cloud collection. Then, principal component analysis (PCA) was used to transform the point cloud into a new coordinate system. The covariance matrix was constructed for the point cloud model, and the eigenvalues and eigenvectors of the covariance matrix were solved. The three eigenvectors were the main directions of the point cloud. According to the centroid coordinates and the principal direction, rotation translation matrix relative to the origin of the initial coordinate system could be established. It transformed the point cloud into the new normalized coordinate system. The corresponding code is expressed in C + + language as:


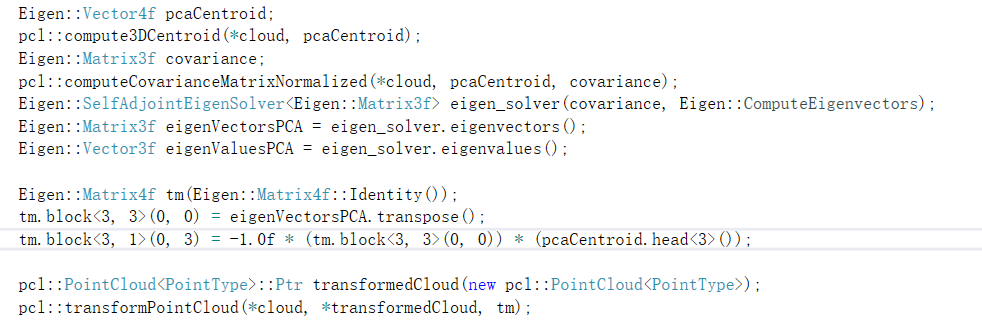


Where cloud was original point cloud and transformedCloud was point cloud after coordinate transformation.

1. **Point cloud filtering and down sampling**

Affected by the scanning environment, the accuracy of the equipment, and the operating habits of experimenters, noise points sometimes appeared in the original point cloud. In view of the characteristics of noise points, statistical filtering was used to denoise point clouds. The corresponding code is expressed in C + + language as:


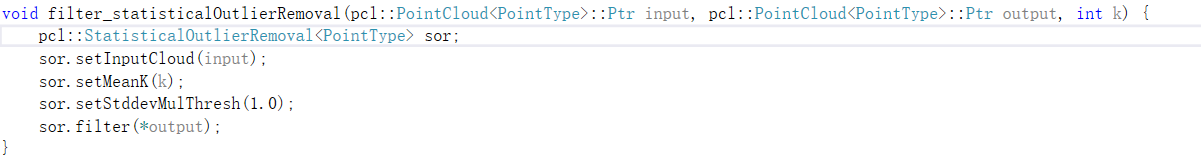


Where statistical coefficient *k* was set to 10 in the code.

Due to the point cloud obtained by the structured light scanner is dense point cloud, the direct processing efficiency was very low. The voxel down sampling of the point cloud could effectively reduce the workload. The corresponding code is expressed in C + + language as:


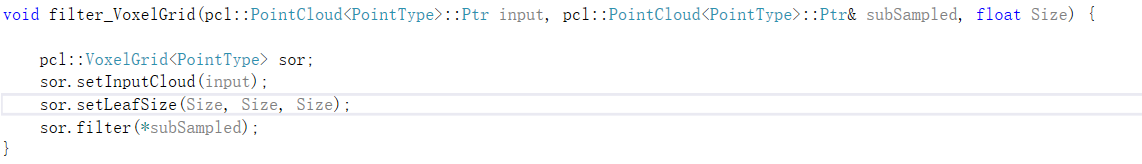


Where voxel side length *Size* was set to 0.25 in the code.

1. **Segmentation of stage and grains**

It was evident that the point cloud of the stage could be regarded as a plane. Therefore, the linear plane model fitting based on the random sample consensus algorithm (RANSAC) was used to estimate the stage plane model. The original point cloud and the plane model were segmented to obtain the stage point cloud and the multiple grains point cloud. The corresponding code is expressed in C + + language as:


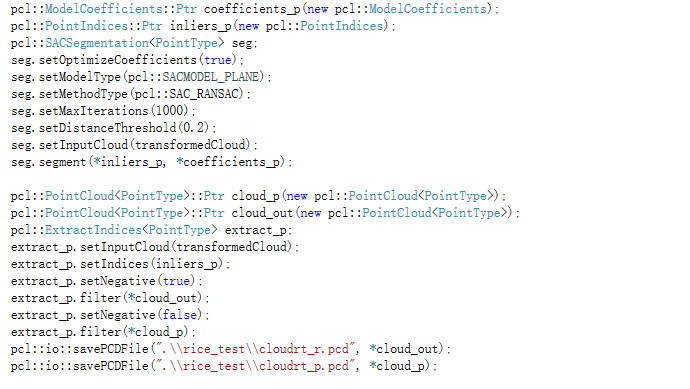


1. **Segmentation of single grains**

After segmenting the stage and grains, the multi-grain point clouds were divided into single grains by region growing algorithm. The point with the smallest curvature value was used as the seed point to create the region growth segmentation object. Then clustering was carried out by the angle and curvature between the normal of seed points and adjacent points. The corresponding code is expressed in C + + language as:


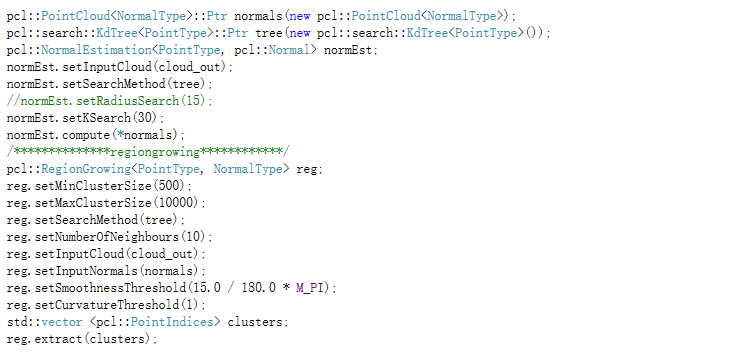


For adapting to rice, wheat and corn grains, minimum cluster point cloud size was set to 500 and maximum cluster point cloud size was set to 10000.

1. **Grain length, width and thickness extraction**

The extraction of grain length, width and thickness was mainly achieved by constructing a bounding box. Firstly, the coordinate system of the segmented single grain point clouds were transformed to convert axis-aligned bounding box (AABB) into orientation bounding box (OBB). Secondly, the maximum and minimum values of the transformed single grain point cloud in the new coordinate system were calculated as $\boldsymbol{x}_{\boldsymbol{max}}、\boldsymbol{x}_{\boldsymbol{min}}、\boldsymbol{y}_{\boldsymbol{max}}、\boldsymbol{y}_{\boldsymbol{min}}、\boldsymbol{z}_{\boldsymbol{max}}、\boldsymbol{z}_{\boldsymbol{min}}$ respectively. Finally, the grain length, width and thickness were computed as following equations.

| $l=x_{max}-x_{min}$ | (1) |
| --- | --- |
| $w=y_{max}-y_{min}$ | (2) |
| $h=z_{max}-z_{min}$ | (3) |

Where l, w and h are the length, width and thickness of a grain, respectively.

The corresponding code is expressed in C + + language as:


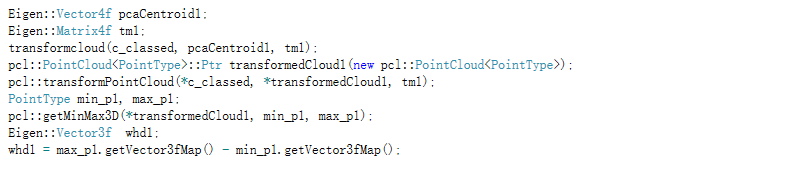


Where *whd1[0]* is thickness, *whd1[1]* is width, *whd1[2]* is length.

1. **Grain surface area extraction**

The triangular mesh model of the point clouds was established by greedy projection triangulation algorithm. It was assumed that the surface of the point cloud was smooth and the density changes were uniform. Point clouds were projected onto a plane based on normal. The projected point cloud was triangulated in the plane. According to the topological connection relation of three points in the plane, a triangular mesh surface model was obtained. Secondly, the holes were filled by reconstructing the mesh boundary edges, which were generated by the grain segmentation. The corresponding code is expressed in C + + language as:


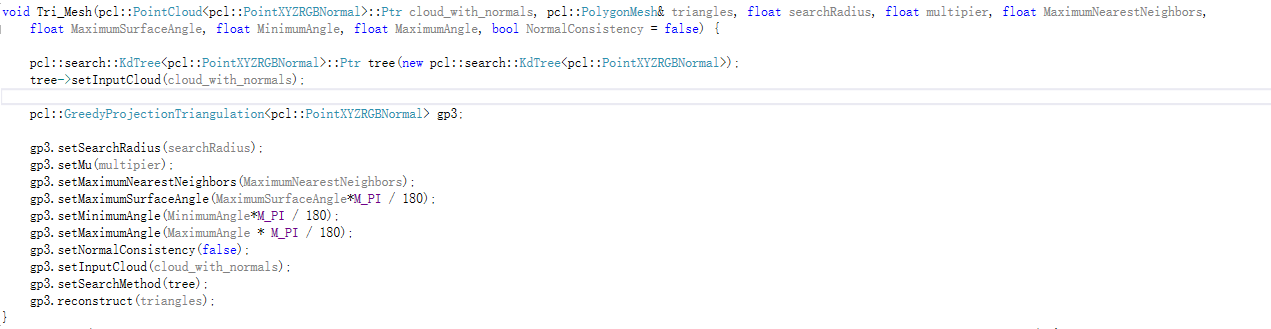


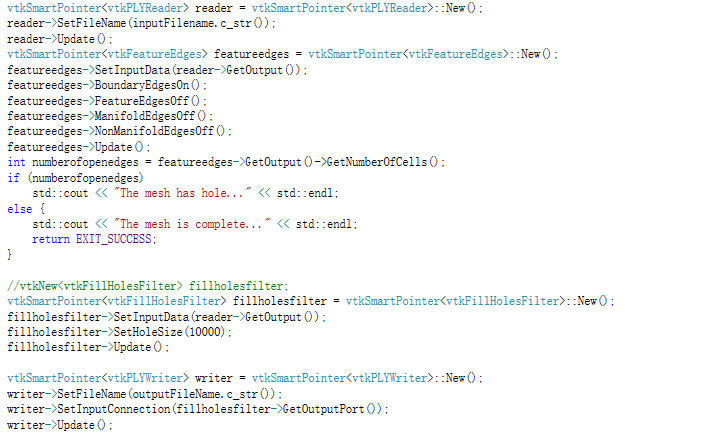


1. **Grain volume extraction**

Firstly, the convex pentahedrons were constructed by the triangular mesh and central plane projection, and then grain volume V was the sum of their volumes. As shown in the Figure 5, $\boldsymbol{A}_{\mathbf{1}}, \boldsymbol{B}_{\mathbf{1}}\mathrm{and}\boldsymbol{C}_{\mathbf{1}}$ are the three vertices of a triangular mesh. It is assumed that the volume of the straight triangular prism $\boldsymbol{A}_{\mathbf{0}}\boldsymbol{B}_{\mathbf{0}}\boldsymbol{C}_{\mathbf{0}}\boldsymbol{ABC}$ is equal to the volume of this convex pentahedron, then the height of the straight triangular prism could be approximated as the height of the gravity center of ${\triangle\boldsymbol{A}}_{\mathbf{1}}\boldsymbol{B}_{\mathbf{1}}\boldsymbol{C}_{\mathbf{1}}$.

| $V_{A_{1}B_{1}C_{1}\mathrm{ABC}}=V_{A_{0}B_{0}C_{0}\mathrm{ABC}}=S_{\triangle ABC}\times h\approx S_{\triangle ABC}\times h_{0}$ | (4) |
| --- | --- |

Where $h$ is height of the straight prism, $h_{0}$ is height of the center of gravity of ${\triangle\boldsymbol{A}}_{\mathbf{1}}\boldsymbol{B}_{\mathbf{1}}\boldsymbol{C}_{\mathbf{1}}$

**Figure 4 Grain volume calculation method**

The corresponding code is expressed in C + + language as:


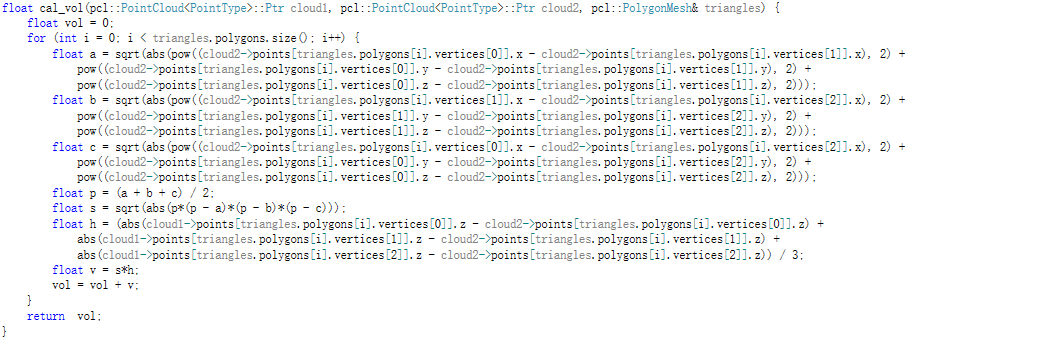


Where cloud1 was grain point cloud, cloud2 was projected point cloud

1. **Projected area and perimeter of grain in the main direction extraction**

In this study, three main directions of grain point cloud were projected, and the projected area and perimeter of cross section, longitudinal section, and horizontal section were obtained as the shape description of grain. Firstly, the point cloud of a single grain after coordinate transformation was projected on the plane of x=0, y=0, z=0 respectively. Then, based on the greedy projection algorithm, the areas of the projected triangular mesh and the perimeter of the mesh edges were calculated. Taking x=0 plane as an example, the corresponding code is expressed in C + + language as:


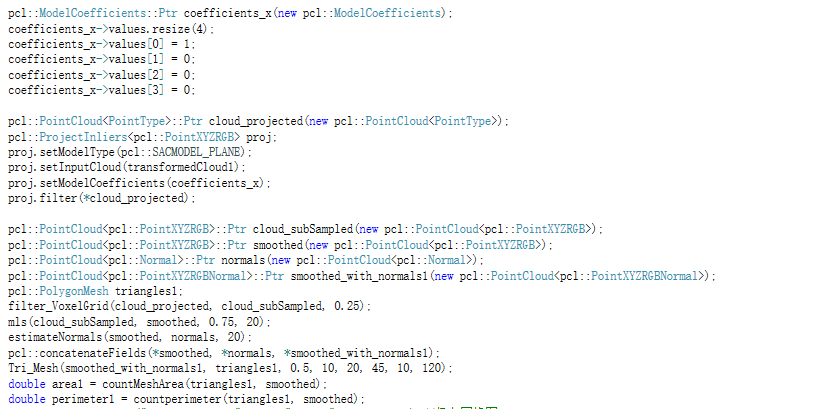


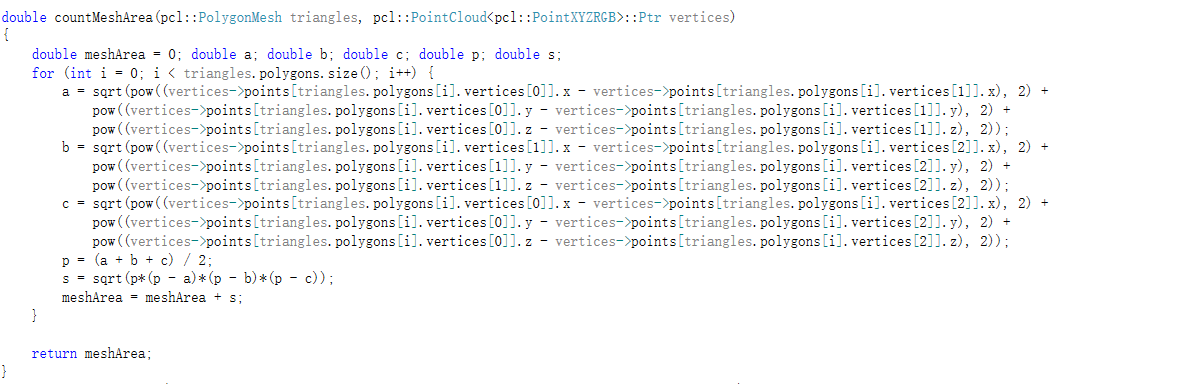


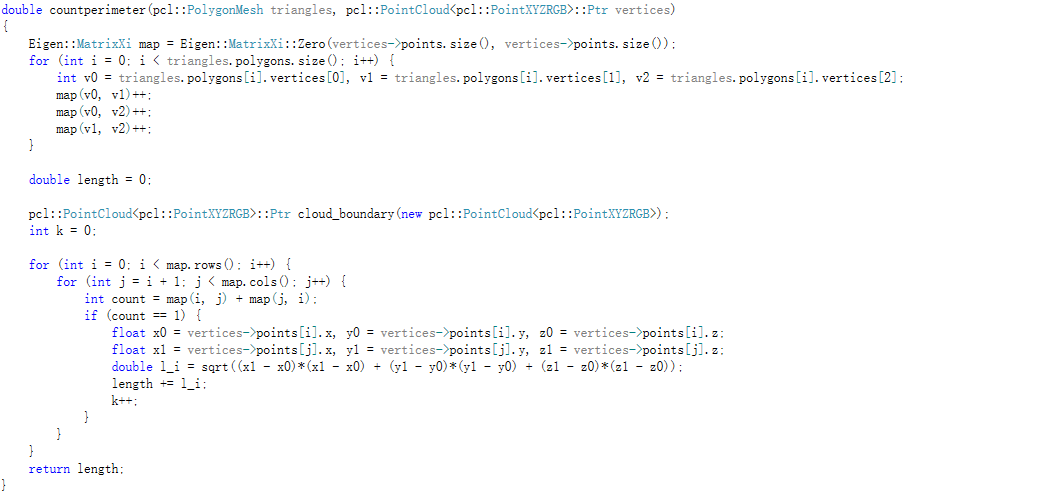


**Algorithm dependency library**

The algorithm is based on cross-platform open source Point Cloud library (PCL) and Visualization Toolkit (VTK). The following are the dependency libraries:

OpenNI2.dll

pcl_common_release.dll

pcl_features_release.dll

pcl_filters_release.dll

pcl_io_ply_release.dll

pcl_io_release.dll

pcl_ml_releasse.dll

pcl_kdtree_release.dll

pcl_octree_ release.dll

pcl_sample_consensus_release.dll

pcl_search_release.dll

pcl_segmentation_release.dll

pcl_surface_release.dll

**Models establishment of recognition**

With the rice grain phenotypic dataset, the models of recognition between filled and unfilled grains, distinction between indica and japonica, and classification of rice grain sub-varieties were established by six different machine learning algorithms including decision tree, random forest, support vector machine, naive bayes, XGBoost, and BP neural network. All the classification models were based on the Sklearn library (Python), and their main parameters were turned by learning curve and grid search. And, the 10-fold cross-validation method was used to test each model.

1. **Classification and regression trees (CART)**

The corresponding code was expressed in Python language as follows. First of all, divide the data set.


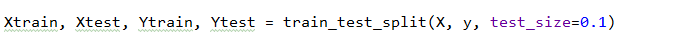


Based on grid search, we could preliminarily determine some parameters.


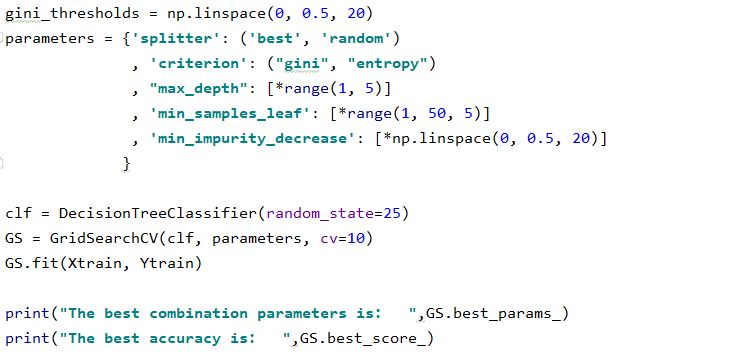


Then single parameter was decided by learning curve.


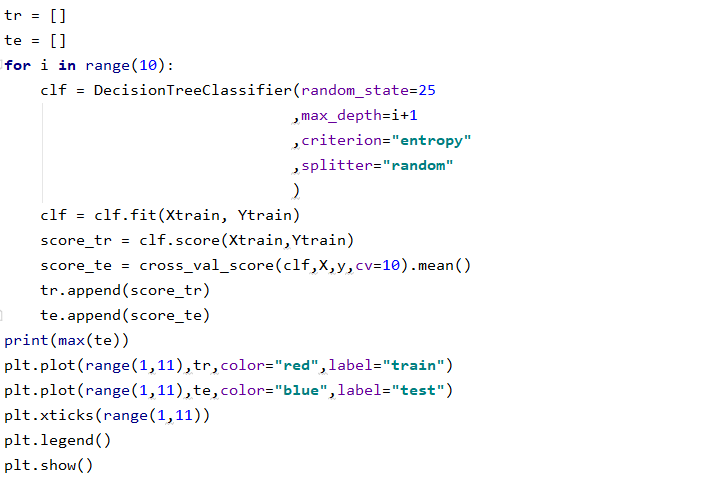


As is shown in Figure 5, *max_depth* was supposed to be 5 for best result.

**
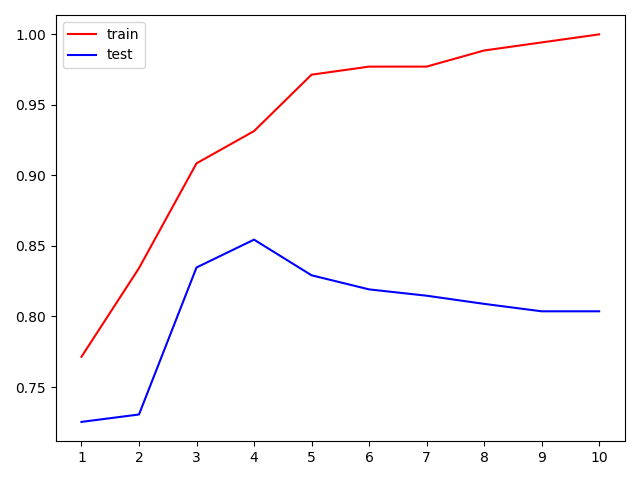
**

**Figure 5 Learning curve of the maximum tree depth**

Finally, based on the determined parameters, the 10-fold cross-validation method was used to test the model for obtaining accuracy, F1 score and AUC value.


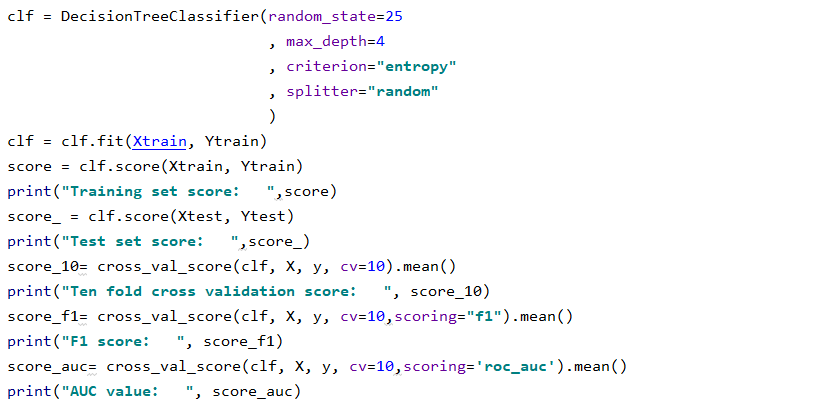


1. **Random forest (RF)**

The validation code was expressed in Python language as follows


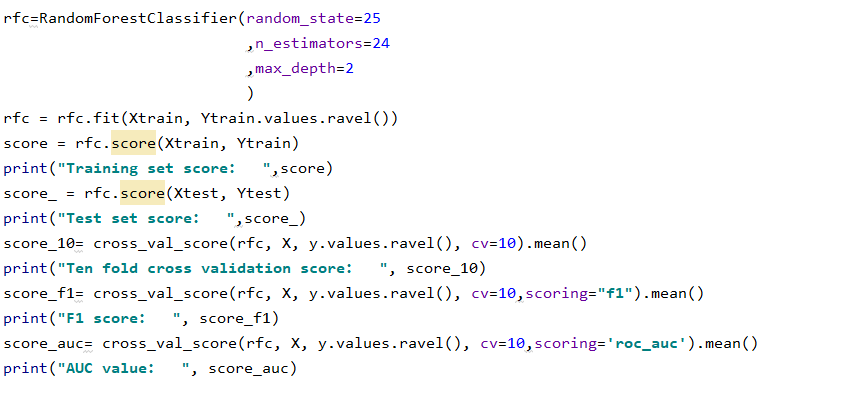


1. **Support vector machines (SVM)**

The validation code was expressed in Python language as follows


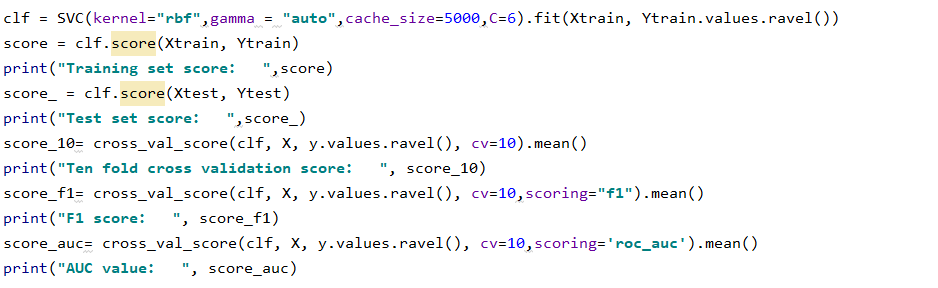


1. **Naive bayes (NB)**

The validation code was expressed in Python language as follows


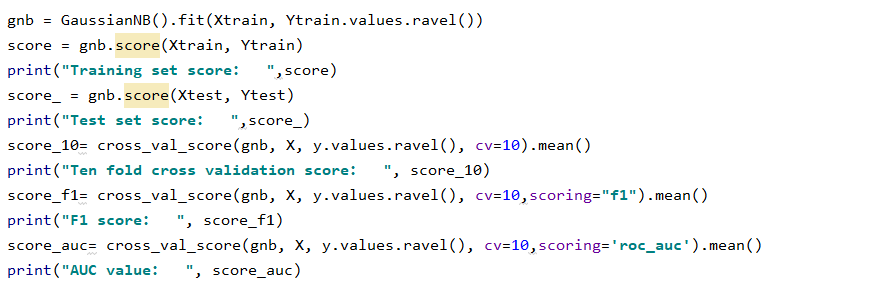


1. **Back propagation (BP)**

The validation code was expressed in Python language as follows


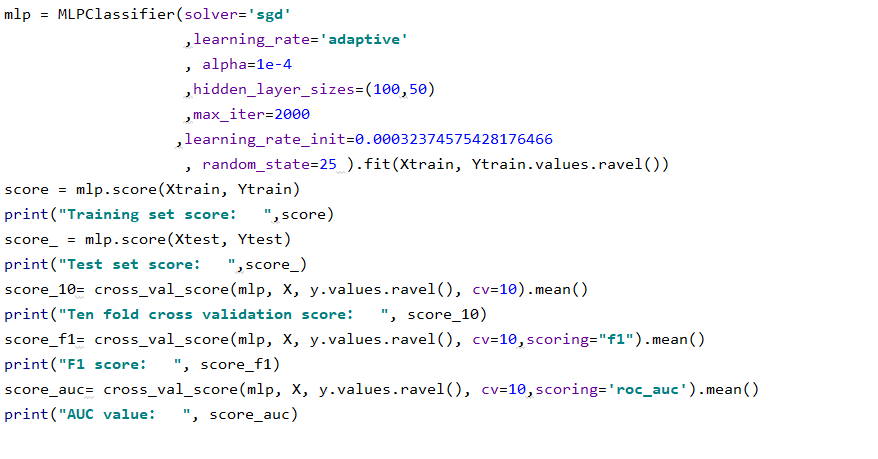


1. **Extreme gradient boosting (XGBoost)**

The validation code was expressed in Python language as follows


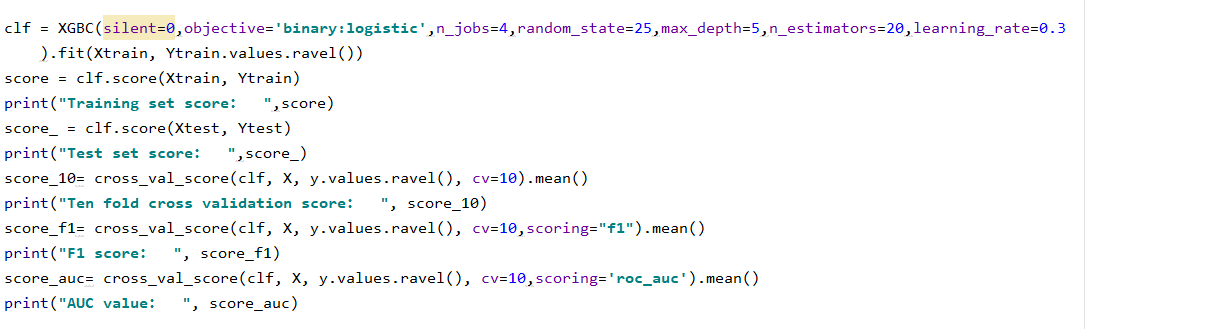


**Contribution of phenotypic parameters**

Comparing the above 6 machine learning methods, XGBoost achieved the best results In order to explore the contribution of phenotypic parameters, the XGBoost classifier was analyzed in detail. The corresponding code was expressed in Python language as follows.


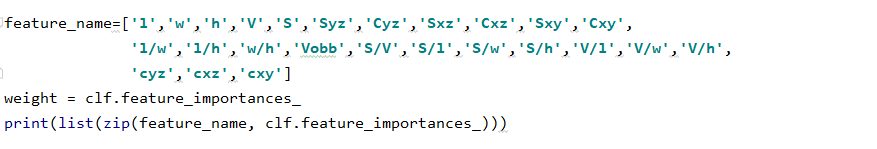


The weight rank of characteristic parameters in XGBoost can refer to the Additional File 6: Appendix S2. And the entire experimental workflow can refer to the Additional File 1 Video S1.
