# Supplementary material for: Cereal grain 3D point cloud analysis method for shape extraction and filled/unfilled grain identification based on structured light imaging
Source: Sci Rep. 2022 Feb 24;12:3145. doi: 10.1038/s41598-022-07221-4 (PMC8873360; doi:10.1038/s41598-022-07221-4)
Supplement: Supplementary file 2 — Supplementary Information 2. [file 41598_2022_7221_MOESM2_ESM.docx]

**Supplementary Tables**

Table A1. Relative error statistics of the grain phenotypic system value and the manual value.

| **No** | **Length** | | | | **Width** | | | | **Thickness** | | | |
| --- | --- | --- | --- | --- | --- | --- | --- | --- | --- | --- | --- | --- |
|  | **Ground truth/mm** | **System /mm** | **RMSE /mm** | **MAPE/%** | **Ground truth /mm** | **System /mm** | **RMSE /mm** | **MAPE /%** | **Ground truth /mm** | **System /mm** | **RMSE /mm** | **MAPE /%** |
| 1 | 7.409 | 7.250 | 0.184 | 2.15 | 3.345 | 3.322 | 0.070 | 0.68 | 2.248 | 2.221 | 0.042 | 1.18 |
| 2 | 7.305 | 7.141 | 0.186 | 2.21 | 3.588 | 3.567 | 0.082 | 0.59 | 2.463 | 2.426 | 0.049 | 1.51 |
| 3 | 7.130 | 6.977 | 0.177 | 2.15 | 3.507 | 3.485 | 0.082 | 0.65 | 2.375 | 2.341 | 0.049 | 1.45 |
| 4 | 7.145 | 6.993 | 0.175 | 2.14 | 3.531 | 3.502 | 0.080 | 0.82 | 2.265 | 2.235 | 0.044 | 1.31 |
| 5 | 7.620 | 7.452 | 0.186 | 2.19 | 3.482 | 3.450 | 0.075 | 0.90 | 2.277 | 2.247 | 0.047 | 1.32 |
| 6 | 9.730 | 9.523 | 0.244 | 2.12 | 2.803 | 2.770 | 0.064 | 1.20 | 2.186 | 2.160 | 0.038 | 1.12 |
| 7 | 10.087 | 9.833 | 0.283 | 2.52 | 2.688 | 2.653 | 0.070 | 1.40 | 2.299 | 2.267 | 0.049 | 1.28 |
| 8 | 9.674 | 9.487 | 0.227 | 1.93 | 2.783 | 2.758 | 0.062 | 0.93 | 2.080 | 2.052 | 0.045 | 1.28 |
| 9 | 9.866 | 9.643 | 0.254 | 2.26 | 2.686 | 2.658 | 0.063 | 0.97 | 2.352 | 2.324 | 0.048 | 1.24 |
| 10 | 10.015 | 9.813 | 0.237 | 2.02 | 2.569 | 2.552 | 0.057 | 0.81 | 2.205 | 2.186 | 0.044 | 0.72 |
| 11 | 7.258 | 7.097 | 0.188 | 2.22 | 3.246 | 3.206 | 0.080 | 1.24 | 1.846 | 1.870 | 0.046 | 1.30 |
| 12 | 6.770 | 6.630 | 0.163 | 2.06 | 3.316 | 3.277 | 0.077 | 1.18 | 2.005 | 2.028 | 0.049 | 1.17 |
| 13 | 6.798 | 6.643 | 0.179 | 2.28 | 3.328 | 3.294 | 0.077 | 1.01 | 1.923 | 1.939 | 0.041 | 0.83 |
| 14 | 6.743 | 6.598 | 0.169 | 2.15 | 3.289 | 3.247 | 0.080 | 1.27 | 1.898 | 1.908 | 0.038 | 0.53 |
| 15 | 6.985 | 6.838 | 0.174 | 2.11 | 3.181 | 3.141 | 0.076 | 1.24 | 1.967 | 1.988 | 0.049 | 1.07 |
| 16 | 8.618 | 8.420 | 0.224 | 2.29 | 2.857 | 2.827 | 0.069 | 1.04 | 1.834 | 1.849 | 0.041 | 0.82 |
| 17 | 8.994 | 8.793 | 0.237 | 2.24 | 2.722 | 2.705 | 0.059 | 0.63 | 1.854 | 1.873 | 0.046 | 0.99 |
| 18 | 8.835 | 8.652 | 0.219 | 2.07 | 2.962 | 2.921 | 0.076 | 1.38 | 1.776 | 1.795 | 0.043 | 1.12 |
| 19 | 9.110 | 8.915 | 0.229 | 2.15 | 2.723 | 2.693 | 0.075 | 1.13 | 1.929 | 1.949 | 0.043 | 1.02 |
| 20 | 9.466 | 9.267 | 0.235 | 2.11 | 2.760 | 2.731 | 0.068 | 1.06 | 1.905 | 1.936 | 0.050 | 1.65 |
| 21 | 6.699 | 6.621 | 0.144 | 1.16 | 3.897 | 3.912 | 0.061 | 0.40 | 3.249 | 3.285 | 0.057 | 1.10 |
| 22 | 12.199 | 12.074 | 0.241 | 1.02 | 8.447 | 8.515 | 0.140 | 0.80 | 5.742 | 5.795 | 0.082 | 0.92 |
| Mean |  |  | 0.210 | 2.07 |  |  | 0.076 | 0.97 |  |  | 0.048 | 1.13 |

**No** is different cereal varieties. 1: Zhonghua 11 filled grain; 2: Wuyunjing 3 filled grain; 3: Nanjing 2728 filled grain, 4: Zhenghan 10 filled grain; 5: Nipponbare filled grain; 6: C Liangyou Huazhan filled grain; 7: Zhuliangyou 211 filled grain; 8: Liangyou 336 filled grain; 9: Fengliangyou No. 4 filled grain; 10: Guangliangyouxiang 66 filled grain; 11: Zhonghua 11 unfilled grain; 12: Wuyunjing 3 unfilled grain; 13: Nanjing 2728 unfilled grain, 14: Zhenghan 10 unfilled grain; 15: Nipponbare unfilled grain; 16: C Liangyou Huazhan unfilled grain; 17: Zhuliangyou 211 unfilled grain; 18: Liangyou 336 unfilled grain; 19: Fengliangyou No. 4 unfilled grain; 20: Guangliangyouxiang 66 unfilled grain; 21: Jimai 22 wheat; 22: Zhengdan 958 corn.

Table A2. Weight rank of characteristic parameters

| Rank | Parameter | Importance weight |
| --- | --- | --- |
| 1 | Thickness | 0.342219 |
| 2 | Length | 0.067255 |
| 3 | Perimeter of horizontal section | 0.062472 |
| 4 | Volume-width ratio | 0.056376 |
| 5 | Compactness index of horizontal section | 0.053502 |
| 6 | Volume | 0.049749 |
| 7 | Length-thickness ratio | 0.042486 |
| 8 | Surface area-length ratio | 0.042199 |
| 9 | Compactness index of cross section | 0.03583384 |
| 10 | Surface area-thickness ratio | 0.034772 |
| 11 | Area of longitudinal section | 0.031442 |
| 12 | Width-thickness ratio | 0.023463 |
| 13 | Area of horizontal section | 0.020139 |
| 14 | Length-width ratio | 0.018671 |
| 15 | Volume-length ratio | 0.018309 |
| 16 | Area of cross section | 0.018093 |
| 17 | Surface area | 0.017622 |
| 18 | Box volume | 0.017193 |
| 19 | Surface area-width ratio | 0.015798701 |
| 20 | Width | 0.014987091 |
| 21 | Compactness index of longitudinal section | 0.011963441 |
| 22 | Volume-thickness ratio | 0.005452804 |
| 23 | Perimeter of cross section | 0 |
| 24 | Perimeter of longitudinal section | 0 |
| 25 | Specific surface area | 0 |

Table A3. 10 rice varieties classification precision result of each classification method

| Classification target | Method | | Precision | Recall score | | F1 score | |  |
| --- | --- | --- | --- | --- | --- | --- | --- | --- |
| Zhonghua 11 | CART | 30.312% | | | 0.39028 | | 0.31640 | |
|  | RF | 36.398% | | | 0.41556 | | 0.35433 | |
|  | SVM | 43.700% | | | 0.45361 | | 0.42594 | |
|  | NB | 36.265% | | | 0.40750 | | 0.37324 | |
|  | BP | 37.898% | | | 0.39722 | | 0.35766 | |
|  | XGBoost | 55.132% | | | 0.48389 | | 0.47640 | |
| Wuyunjing 3 | CART | | 34.478% | 0.38306 | | 0.32017 | |  |
|  | RF | | 32.465% | 0.38194 | | 0.32344 | |  |
|  | SVM | | 58.310% | 0.59389 | | 0.56631 | |  |
|  | NB | | 42.113% | 0.43167 | | 0.40698 | |  |
|  | BP | | 40.134% | 0.41389 | | 0.38438 | |  |
|  | XGBoost | | 45.019% | 0.47750 | | 0.45777 | |  |
| Nanjing 2728 | CART | | 39.699% | 0.40750 | | 0.35274 | |  |
|  | RF | | 35.166% | 0.38611 | | 0.34244 | |  |
|  | SVM | | 49.661% | 0.48333 | | 0.46824 | |  |
|  | NB | | 56.490% | 0.53056 | | 0.52422 | |  |
|  | BP | | 43.697% | 0.42861 | | 0.42385 | |  |
|  | XGBoost | | 44.260% | 0.44722 | | 0.43703 | |  |
| Zhenghan 10 | CART | | 33.907% | 0.39389 | | 0.33783 | |  |
|  | RF | | 50.030% | 0.51139 | | 0.47413 | |  |
|  | SVM | | 55.742% | 0.57056 | | 0.54662 | |  |
|  | NB | | 57.518 % | 0.57806 | | 0.55702 | |  |
|  | BP | | 45.359% | 0.45139 | | 0.43532 | |  |
|  | XGBoost | | 52.349% | 0.51889 | | 0.50953 | |  |
| Nipponbare | CART | | 45.099% | 0.45417 | | 0.41690 | |  |
|  | RF | | 31.651% | 0.39389 | | 0.33019 | |  |
|  | SVM | | 59.157% | 0.53639 | | 0.53299 | |  |
|  | NB | | 52.572% | 0.48556 | | 0.47640 | |  |
|  | BP | | 36.410% | 0.34694 | | 0.33999 | |  |
|  | XGBoost | | 51.714% | 0.48583 | | 0.48958 | |  |
| C Liangyou Huazhan | CART | | 28.989% | 0.34250 | | 0.28647 | |  |
|  | RF | | 35.313% | 0.39222 | | 0.33595 | |  |
|  | SVM | | 47.291% | 0.41917 | | 0.39486 | |  |
|  | NB | | 36.403% | 0.36611 | | 0.33759 | |  |
|  | BP | | 39.110% | 0.35750 | | 0.34339 | |  |
|  | XGBoost | | 53.389% | 0.49722 | | 0.48416 | |  |
| Zhulaingyou 211 | CART | | 18.568% | 0.29333 | | 0.22038 | |  |
|  | RF | | 34.818% | 0.40778 | | 0.34583 | |  |
|  | SVM | | 41.369% | 0.41361 | | 0.38233 | |  |
|  | NB | | 33.516% | 0.37167 | | 0.33518 | |  |
|  | BP | | 34.395% | 0.35861 | | 0.34067 | |  |
|  | XGBoost | | 54.304% | 0.47694 | | 0.47629 | |  |
| Liangyou 336 | CART | | 29.382% | 0.36559 | | 0.30665 | |  |
|  | RF | | 28.997% | 0.36111 | | 0.30898 | |  |
|  | SVM | | 43.593% | 0.41944 | | 0.40955 | |  |
|  | NB | | 32.009% | 0.34750 | | 0.32184 | |  |
|  | BP | | 35.500% | 0.37250 | | 0.33661 | |  |
|  | XGBoost | | 41.429% | 0.39846 | | 0.40944 | |  |
| Fengliangyou No.4 | CART | | 23.133% | 0.27472 | | 0.23294 | |  |
|  | RF | | 27.186% | 0.31889 | | 0.26557 | |  |
|  | SVM | | 35.127% | 0.35667 | | 0.33438 | |  |
|  | NB | | 30.331% | 0.25722 | | 0.26131 | |  |
|  | BP | | 40.497% | 0.38889 | | 0.38670 | |  |
|  | XGBoost | | 38.286% | 0.35694 | | 0.35331 | |  |
| Guangliangyouxiang 66 | CART | | 25.788% | 0.34528 | | 0.28739 | |  |
|  | RF | | 35.105% | 0.37167 | | 0.34018 | |  |
|  | SVM | | 48.034% | 0.43889 | | 0.42348 | |  |
|  | NB | | 36.680% | 0.34167 | | 0.32344 | |  |
|  | BP | | 30.972% | 0.28917 | | 0.28276 | |  |
|  | XGBoost | | 43.764% | 0.41528 | | 0.41417 | |  |
| Average | CART | | 30.936% | 0.36586 | | 0.30779 | |  |
|  | RF | | 34.713% | 0.39406 | | 0.34210 | |  |
|  | SVM | | 48.199% | 0.46856 | | 0.44847 | |  |
|  | NB | | 41.390% | 0.41175 | | 0.39172 | |  |
|  | BP | | 38.397% | 0.38047 | | 0.36313 | |  |
|  | XGBoost | | 47.695% | 0.45692 | | 0.44967 | |  |
