# Supplementary material for: Cereal grain 3D point cloud analysis method for shape extraction and filled/unfilled grain identification based on structured light imaging
Source: Sci Rep. 2022 Feb 24;12:3145. doi: 10.1038/s41598-022-07221-4 (PMC8873360; doi:10.1038/s41598-022-07221-4)
Supplement: Supplementary file 3 — Supplementary Information 3. [file 41598_2022_7221_MOESM3_ESM.pdf]

Supplementary Information

Supplementary Video S1. The software operation for grain 3D point cloud analysis.wmv

Supplementary Appendix S1. Cereal grain 3D point cloud analysis method technical documentation

Supplementary Appendix S2. Supplementary Tables: Table A1. Relative error statistics of the grain phenotypic system value and the manual value. Table A2. Weight rank of characteristic traits. Table A3. 10 rice varieties classification precision result of each classification method.

Supplementary Table S1. The system value for the 2200 samples including rice, wheat and corn grain.

Supplementary Table S2. The ground truth for the 2200 samples including rice, wheat and corn grain.

Supplementary Table S3. The system value for the Zhonghua 11 filled grain of the horizontal placement.
